# Supplementary material for: R-Loops Promote Antisense Transcription across the Mammalian Genome
Source: Mol Cell. 2019 Nov 21;76(4):600–616.e6. doi: 10.1016/j.molcel.2019.10.002 (PMC6868509; doi:10.1016/j.molcel.2019.10.002)
Supplement: Document S1. Figures S1–S7 and Tables S1–S6 [file mmc1.pdf]

**Molecular Cell, Volume *76***

## Supplemental Information

# R-Loops Promote Antisense Transcription across the Mammalian Genome

**Sue Mei Tan-Wong, Somdutta Dhir, and Nick J. Proudfoot**

# Figure S1 (related to Figure 1)

## A Efficiency of R-loop plasmid immunoselection

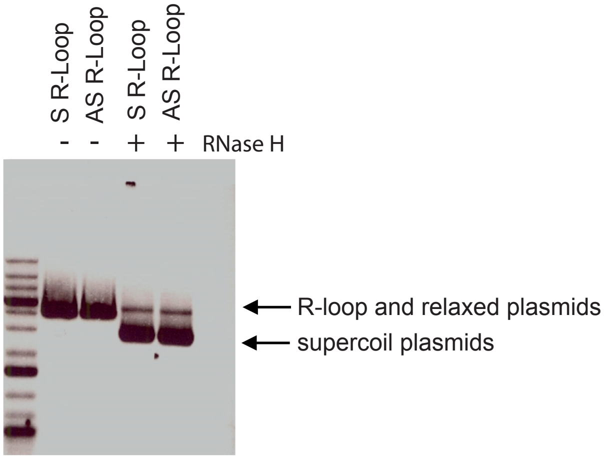


## B Positions of capped AS transcripts

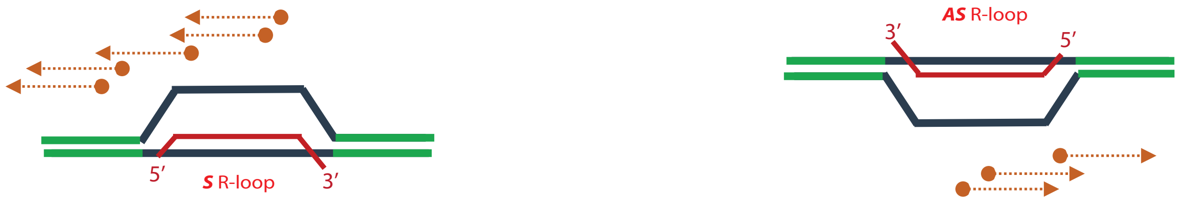


5′ end of AS transcript from S R-loop transcription

GTTGAGCATTAGGTCCCAAAATTTGTTTACTAAAAACACATGTGGATATCTTGACTGATTTTT
CCATGGAGGGCACAGTTAAGCCGCTAAAGGCATTATCCGCCAAGTACAATTTTTTACTCTTC
GAAGACAGAAAATTTGCTGACATTGGTAATACAGTCAAATTGCA**G**TACTCTGCGGGTGTATA
CAGAATAGCAGAATGGGCAGACATTACGAATGCACACGGTGTGGTGGGCCCAGGTATTGTT
AG**C**GGTTTGAAGCAGGCGGCAGAAGAAGTAACAAAGGAACCTAGAGGGGGACTATTTGGG
GGTGTCTGGCT**C**AGGTGCCATGCCTCACTGGGGCTGGTTGGCACCTGCATTTCCTGGAGTGG
GGCTGTCTCAGGGTAGCTGGG**C**ACGGTGTT**C**CCTTGAGTGGGGGTGTAGTGGGTGTTCCTA
GCTGCCACGCCTTTGCCTTCACCTATGGGACCTTTTGATGTTAGCAGAATTGTCATGCAAGGG
CTCCCTATCTACTGGAGAATATACTAAGGGTACTGTTGACATTGCGAAGAGCGACAAAGATT
TTGTTATCGGCTTTATTGCTCAAAGAGACATGGGTGGAAGAGATGAAGGTTACGATTGGTTG
ATTATGACACCCGGTGTGGGTTTAGATGACAAGGGAGACGCATTGGGTCAACAGTATAGAA
CCGTGGATGATGTGGTCTCTACAGGATCTGACATT

5′ end of S transcript from AS R-loop transcription

GTTGAGCATTAGGTCCCAAAATTTGTTTACTAAAAACACATGTGGATATCTTGACTGATTTTT
CCATGGAGGGCACAGTTAAGCCGCTAAAGGCATTATCCGCCAAGTACAATTTTTTACTCTTC
GAAGACAGAAAATTTGCTGACATTGGTAATACAGTCAAATTGCAGTACTCTGCGGGTGTATA
CAGAATAGCAGAATGGGCAGACATTACGAATGCACACGGTGTGGTGGGCCCAGGTATTGTT
AGCGGTTTGAAGCAGGCGGCAGAAGAAGTAACAAAGGAACCTAGAGGGGGACTATTTGGG
GGTGTCTGGCTCAGGTGCCATGCCTCACTGGGGCTGGTTGGCACCTG**C**ATTTC**C**TGGAGTGG
GGCTGTCTCAGGGTAGCTGGGCACGGTGTT**C**CCTTGAGTGGGGGTGTAGTGGGTGTTCCTA
GCTGCCACGCCTTTGCCTTCACCTATGGGACCTTTTGATGTTAGCAGAATTGTCATGCAAGGG
CTCCCTATCTACTGGAGAATATACTAAGGGTACTGTTGACATTGCGAAGAGCGACAAAGATT
TTGTTATCGGCTTTATTGCTCAAAGAGACATGGGTGGAAGAGATGAAGGTTACGATTGGTTG
ATTATGACACCCGGTGTGGGTTTAGATGACAAGGGAGACGCATTGGGTCAACAGTATAGAA
CCGTGGATGATGTGGTCTCTACAGGATCTGACATT

## C Efficiency of R-loop promoter vs Adenovirus major late promoter

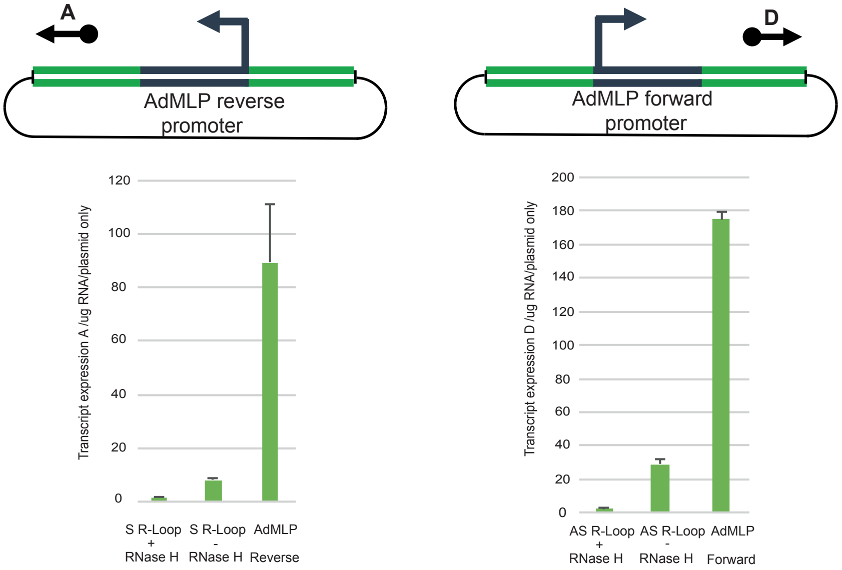


**Figure S1 (related to Figure 1)**
(A) Efficiency of R-loop plasmid immunoselection. Ethidium bromide stained agarose gel of S9.6 immunoselected R-loop plasmids, showing sensitivity to RNase H1 digestion.
(B) Sequence analysis of R-loop induced transcripts. 5'RACE shows different transcription start sites from R-loop promoter, depicted as bold orange nucleotides within the β-actin terminator region (blue nucleotides) or adjacent *URA3* regions (green nucleotides).
(C) Comparison of R-loop and adenovirus major late promoters *in vitro* transcription efficiencies. AdMLP promoter was inserted in place of the β-actin terminator R-loop region in an orientation specific manner within pCIUBU. *In vitro* transcription followed by RT-qPCR was carried out using the same *URA3* gene primer pairs as in Figure 1D. Data represented as mean ± SEM.

# Figure S2 (related to Figure 2)

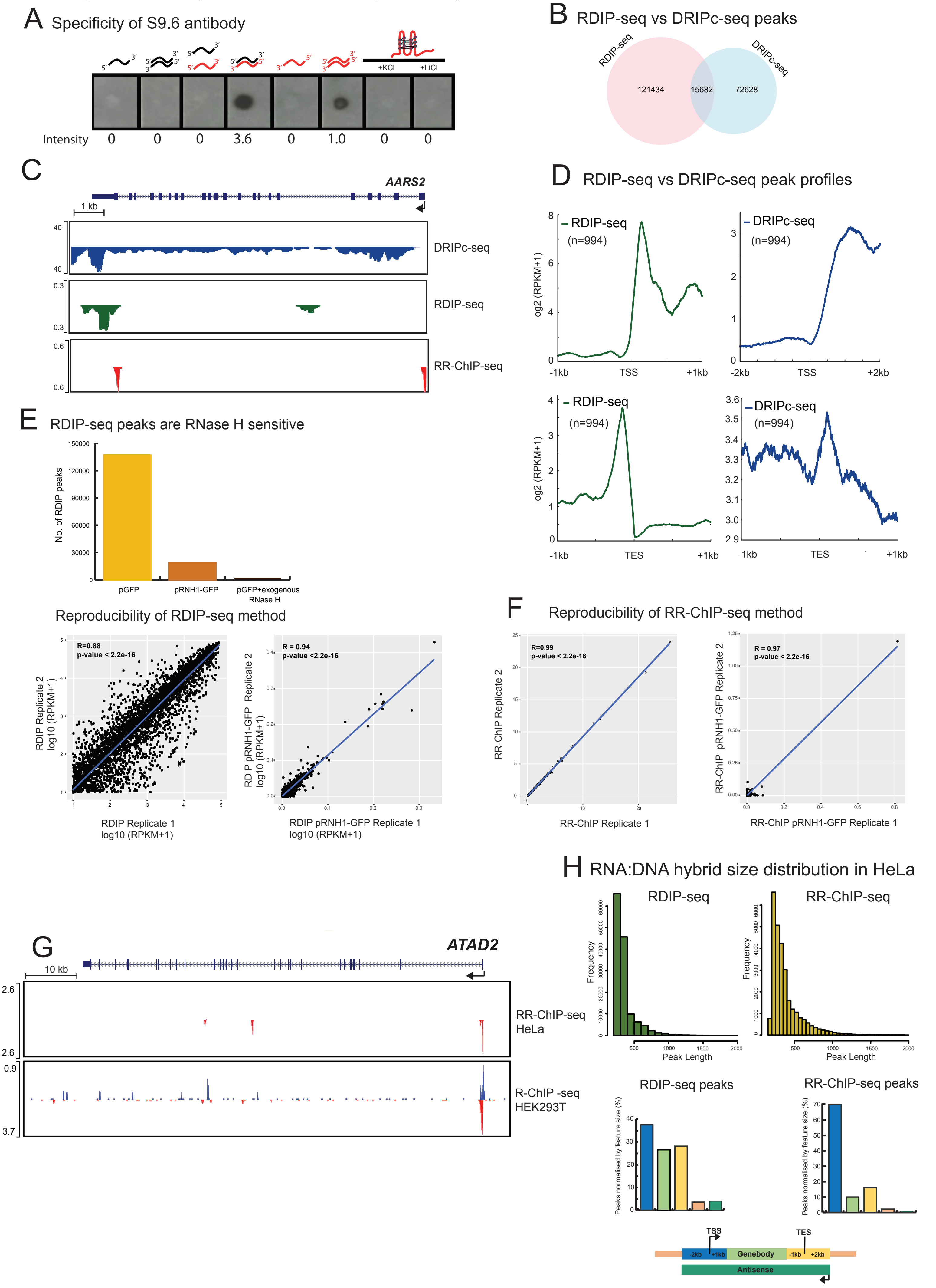

**Figure S2 (related to Figure 2)**

(A) Dot blot analysis of S9.6 antibody binding to different nucleic acid structures as indicated (Table S3). Intensities were measured with ImageStudio Lite program.

(B) Venn diagram showing the overlap of R-loop peaks identified by DRIPc-seq and RDIP-seq.

(C) *AARS2* R-loop profiles for DRIPc-seq, RDIP-seq and RR-ChIP-seq.

(D) Peak density plot showing distribution of RDIP-seq and DRIPc-seq relative to TSS and TES regions of protein coding genes.

(E) Graph showing the high proportion of RDIP-seq peaks that display RNase H sensitivity either by RNase H1 over-expression or by RNase H treatment following the immuno-precipitation step. Below shows a scatter plot of reproducibility between R-loop peaks detected by two RDIP-seq (Pearson correlation: R = 0.88). Transcripts of ≥1 RPKM were analysed. Similarly, for the two RDIP-seq with RNase H1 over-expression.

(F) Scatter plot showing high reproducibility between RR-ChIP-seq and RR-ChIP-seq with RNase H1 over-expression replicates (Pearson correlation: R = 0.99 and 0.97 respectively).

(G) *ATAD2* RR-ChIP-seq vs R-ChIP-seq peak profiles.

(H) Size distribution of RDIP-seq and RR-ChIP-seq peaks in HeLa cells. Level of RDIP-seq and RR-ChIP-seq peaks present at different gene regions, corrected for genomic feature size (lower diagram).

# Figure S3 (related to Figure 3)

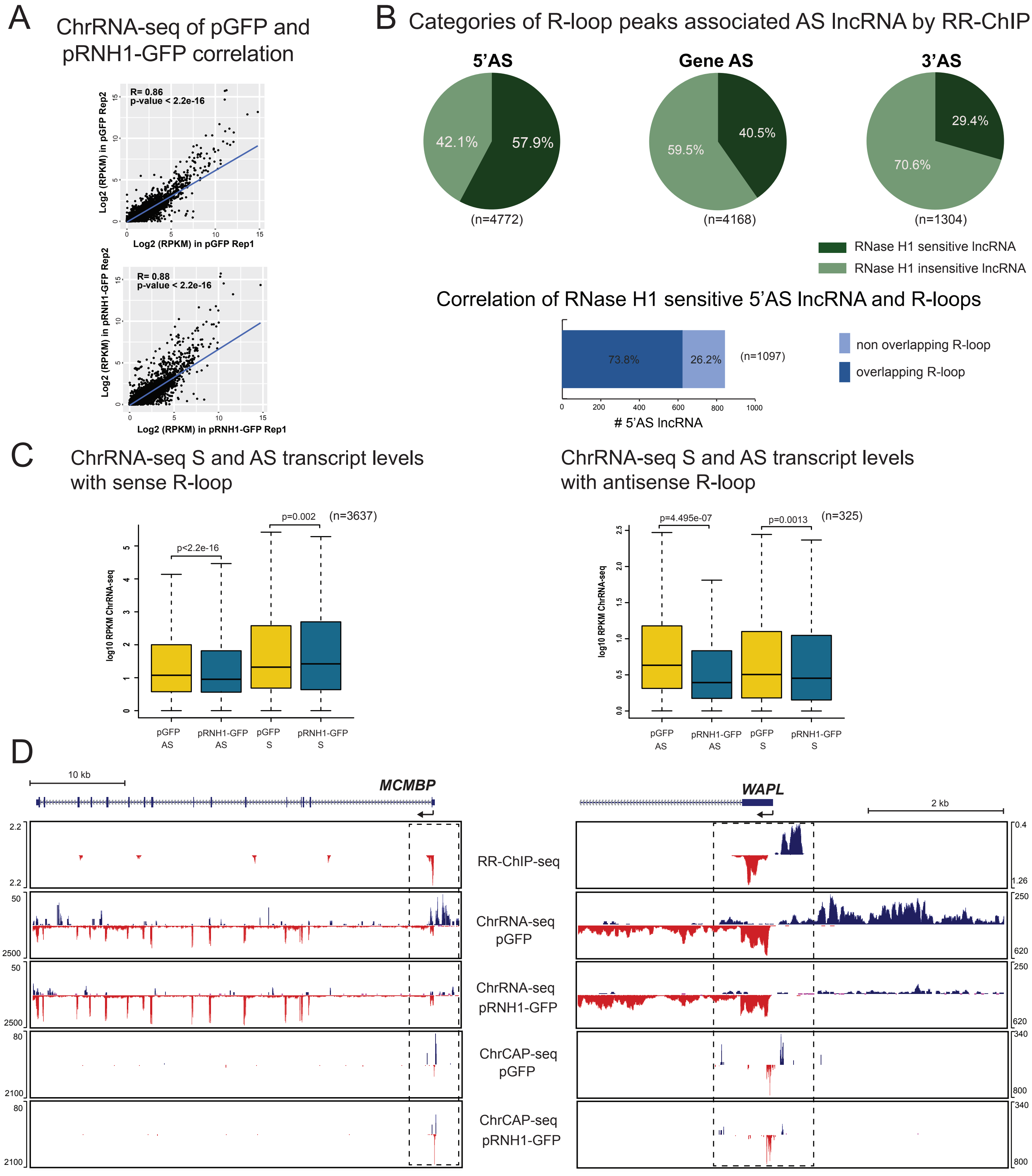


**Figure S3 (related to Figure 3)**

(A) Scatterplot of gene expression showing reproducibility (Pearson correlation: R = 0.86 and 0.88 respectively) between the two ChrRNA-seq datasets for pGFP and pRNH1-GFP over-expression. Transcripts of ≥1 RPKM were used.

(B) Pie diagrams showing total numbers of RR-ChIP peaks (+/-250 bp from lncRNA) for the three AS categories. % of these R-loop associated lncRNA that display RNase H sensitivity is indicated. Stacked bar chart showing correlation between RNase H1 sensitive 5'AS lncRNA and associated pRNH1-GFP sensitive R-loop peaks. 73.8% of RNase H1 sensitive 5'AS lncRNA has a corresponding RNase H1 sensitive R-loop overlap.

(C) Box plots showing range of S gene and AS lncRNA transcript levels associated with sense R-loops (left hand side) or antisense R-loops (right hand side). *P*-values were computed using Wilcoxon test.

(D) *MCMBP* and *WAPL* ChrRNA-seq versus ChrCAP-seq profiles +/- RNase H1 over-expression. RR-ChIP-seq profile also shown.

# Figure S4 (related to Figure 4)

Further eRNAs

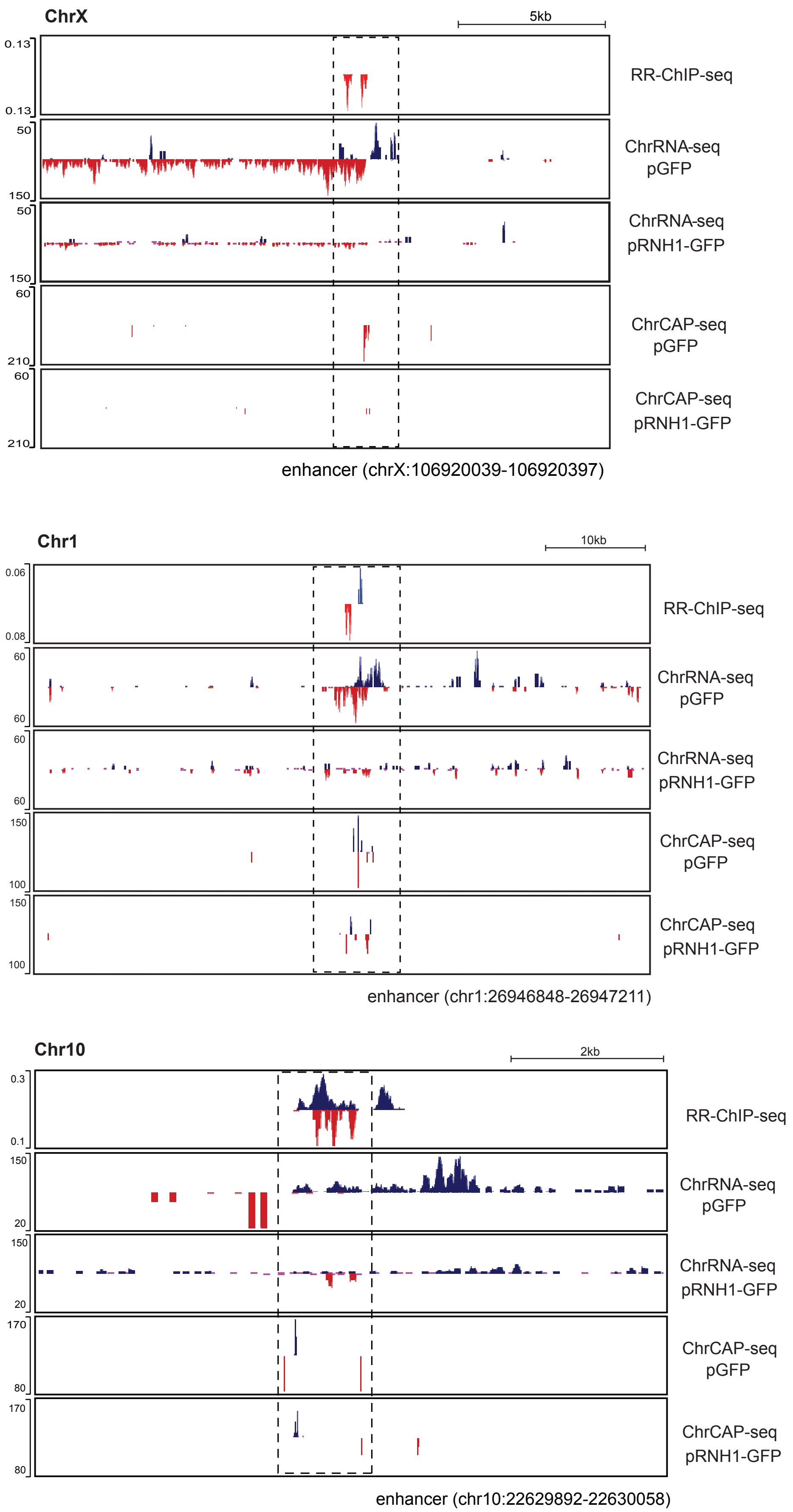


**Figure S4 (related to Figure 4)**
Three separate enhancer-derived eRNA profiles (from chromosome X, 1 and 10) based on ChrRNA-seq and ChrCAP-seq +/- RNase H1 over-expression versus RR-ChIP-seq profiles.

# Figure S5 (related to Figure 5)

A ChrCAP-seq vs 5' GRO-seq comparison in HeLa

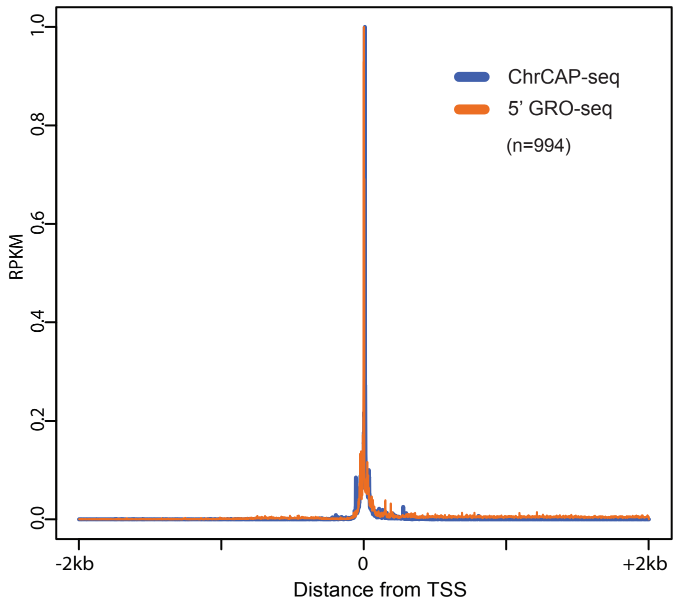


B Comparison example between ChrCAP-seq and 5' GRO-seq

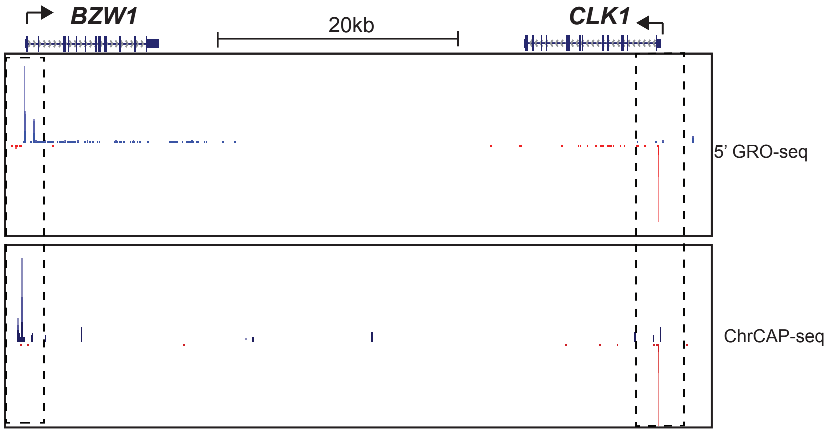


**Figure S5 (related to Figure 5)**
(A) Aggregation plots comparing signals for ChrCAP-seq and 5'GRO-seq from Hela cells across all transcription start sites (TSS) within a 2kb window on either side of TSS.
(B) ChrCAP-seq versus 5'GRO-seq profiles for *BZW1-CLK1* convergent gene locus.

# Figure S6 (related to Figure 6)

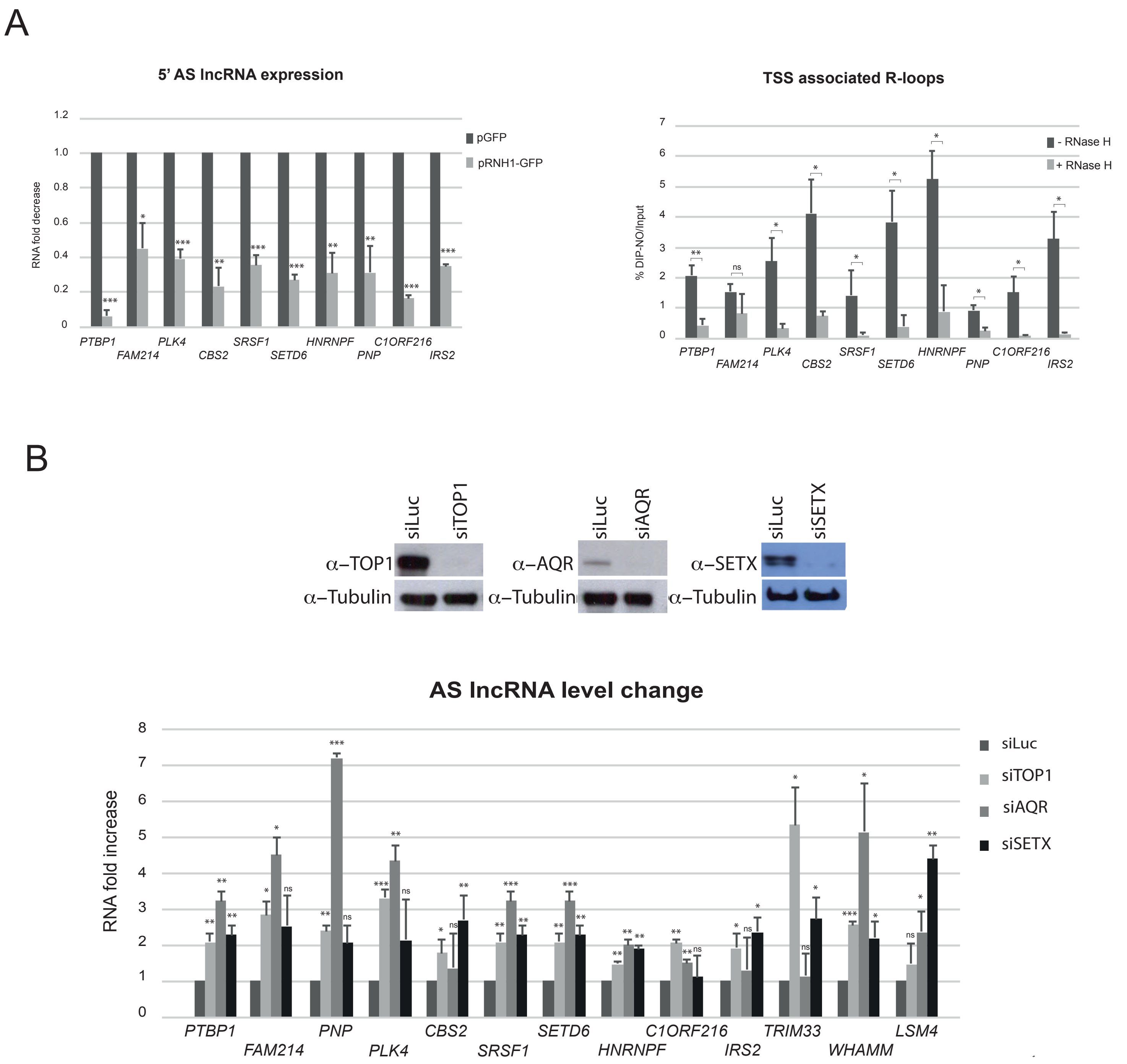


**Figure S6 (related to Figure 6)**
(A) RT-qPCR analysis of specific genes validating expression of RNase H1 sensitive antisense transcripts, identified by ChrRNA-seq. Chromatin RNA fraction from HeLa cells with or without RNase H1 over-expression analyzed using gene specific primers for RT reaction (Table S4). Relative expression values were normalized to 18S RNA. Data represented as mean ± SEM (left). DIP analyses (Skourti-Stathaki et al., 2011) of same regions as above, validating R-loop occupied regions. Data represented as mean ± SEM (right). (n=3, ****p* < 0.001, ***p* < 0.01 and **p* < 0.05, paired *t*-test).
(B) Enhanced R-loop formation increases antisense transcript levels. Top panels are western blots showing efficiency of TOP1, AQR and SETX knock downs following siRNA treatment. RT-qPCR results show that stabilizing R-loops by the various factor depletions increases antisense transcript levels for various genes. Data represented as mean ± SEM (n=3, ****p* < 0.001, ***p* < 0.01 and **p* < 0.05, paired *t*-test).

# Figure S7 (related to Figure 7)

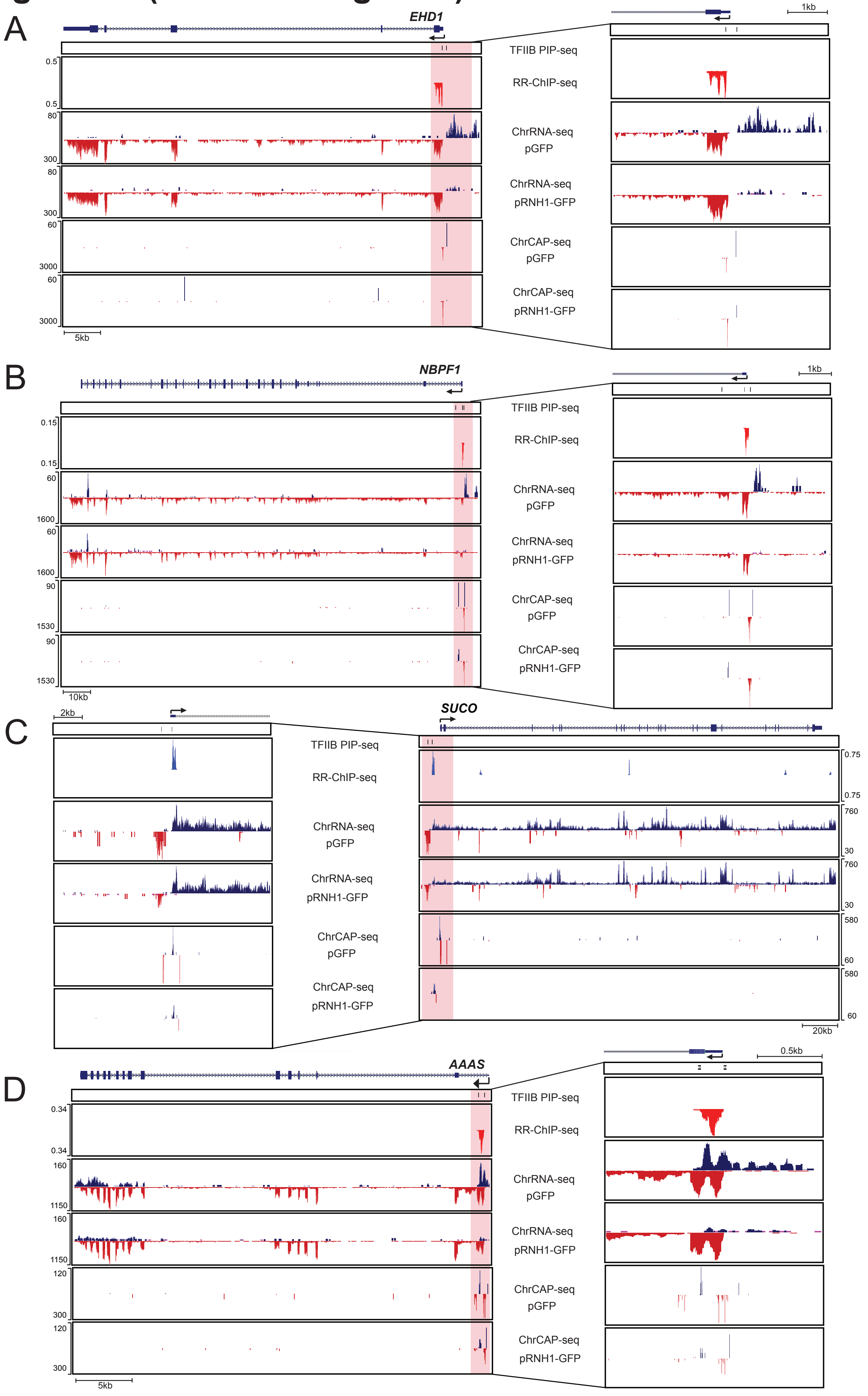


**Figure S7 (related to Figure 7)**
ChrRNA-seq and ChrCAP-seq profiles +/- RNase H1 over-expression compared to TFIIB-PIP-seq and RR-ChIP-seq for 4 selected genes as in Figure 7. Promoter regions are highlighted with pink shading and shown enlarged.

**Table S1: Primers for plasmid pCIUBU construction and strand specific RNA transcript (related to STAR Methods)**

| PRIMER'S NAME | PRIMER'S SEQUENCES (5'-3') |
|---|---|
| *H.s.* ACTB terminator Forward | GGGACTATTTGGGGGTGTCTGGCT |
| *H.s.* ACTB terminator Reverse | CGATCCCATAGGTGAAGGCAAAGG |
| *S. c. URA3* Forward | GATTCGGTAATCACCGAACAG |
| *S. c. URA3* Reverse | GGAATCTCGGTCGTAATGATT |
| T7 *H.s.* ACTB terminator Forward | TTAATACGACTCACTATAGGGAGAGGACTATTTGGGGGTGTCTGGCT |
| T3 *H.s.* ACTB terminator Reverse | AATTAACCCTCACTAAAGGGAGACGATCCCATAGGTGAAGGCAAAGG |

**Table S2: Primers for RT-qPCR for *in vitro* transcription (related to STAR Methods)**

| PRIMER'S NAME | PRIMER'S SEQUENCES (5'-3') |
|---|---|
| Amplicon A Forward | ATAATGCCTTTAGCGGCTTA |
| Amplicon A Reverse | AACTTGTGTGCTTCATTGGA |
| Amplicon B Forward | AACTTGTGTGCTTCATTGGA |
| Amplicon B Reverse | ATAATGCCTTTAGCGGCTTA |
| Amplicon C Forward | GCTTCCCAGCCTGCTTTTCTG |
| Amplicon C Reverse | TAGATGACAAGGGAGACGCAT |
| Amplicon D Forward | TAGATGACAAGGGAGACGCAT |
| Amplicon D Reverse | GCTTCCCAGCCTGCTTTTCTG |
| Amplicon A RT primer | CATGCACGAAAAGCAAACAAAC |
| Amplicon B RT primer | TTGTACTTGGCGGATAATGCC |
| Amplicon C RT primer | ACACCCGGTGTGGGTTTAGAT |
| Amplicon D RT primer | CATCTTCTCAAATATGCTTCC |

**Table S3: Sequences of DNA or RNA oligonucleotides used for S9.6 validation on dot blot (related to STAR methods)**

| NUCLEOTIDE NAME | NUCLEOTIDE SEQUENCES |
|---|---|
| DNA 5' to 3' | GTTCAGAGTTCTACAGTCCGACGATC |
| DNA 3' to 5' | GATCGTCGGACTGTAGAACTCTGAAC |
| RNA 5'to 3' | rGUUrCrArGrArGUUrCUrArCrArGUrCrCrGrArCrGrAUrC |
| RNA 3'to 5' | rGrAUrCrGUrCrGrGrArCUrGUrArGrArArCUrCUrGrArArC |
| RNA G-quadruplex | $(GAGCUGGGGU)_4$ |

**Table S4: Primers for RT-qPCR of antisense transcript, Br-U NRO and DIP validations (related to STAR methods)**

| PRIMER'S NAME | PRIMER'S SEQUENCES (5'-3') |
|---|---|
| TRIM33 for AS forward | CCCTCGAAGCCAGTTCCTTG |
| TRIM33 for AS reverse | GGCTACCACCCTTCCGTATG |
| TRIM33 for DIP forward | ATGCCCAGCTTTCCCTAACT |
| TRIM33 for DIP reverse | GGAAAGTGGACTGCATGGTT |
| WHAMM for AS forward | CAGACTCCTGAACAGGTTGC |
| WHAMM for AS reverse | GCTTACAAGAGGCATTCGG |
| WHAMM for DIP forward | GACGCGGTTTCGATTCTAGC |
| WHAMM for DIP reverse | AGGCTGTCAGGCTGCTCGTC |
| LSM4 for AS and DIP forward | TTGAGAGTCTCGATCTGTCG |
| LSM4 for AS and DIP reverse | TGATGGCACAAACCTGTAATC |
| MYOD1 forward | CGCTTTCCTTAACCACAAATC |
| MYOD1 reverse | AACACGGTCGTCATAGAAGT |
| SRSF1 for AS forward | CGAATAGACCGTGGCTGCTT |
| SRSF1 for AS reverse | GAGTGCTGACCCGAGCTATG |
| SRSF1 for DIP forward | GGTCCTCGAACTCAACGAAG |
| SRSF1 for DIP reverse | CGCATCTACGTGGGTAACTTA |

| | |
|---|---|
| PLK4 for AS forward | GAGGTCTCTTTTGATCCATACAA |
| PLK4 for AS reverse | AACACCGTTTACACCACTACC |
| PLK4 for DIP forward | GAAGGTGTCAGGGAGAACTTT |
| PLK4 for DIP reverse | CTGCCGAGTCTTTTCACCTC |
| SEDT6  for AS forward | CGGTCTGGGTGCAAGAACAA |
| SEDT6 for AS reverse | GCAACAAGGGGTCGAGATCA |
| SEDT6 for DIP forward | CTGCTTCCTGAGCTGGTGC |
| SEDT6 for DIP reverse | CACCACGAACAACAGCTCT |
| FAM214A for AS forward | CAGACTACAGGCATGCACAA |
| FAM214A for AS reverse | GCTCACTTTATGAATACAAGGCT |
| FAM214A for DIP forward | CAGGCTGTAAACACCTCCC |
| FAM214A for DIP reverse | GGCCAAGTTTACACAGAAACC |
| HNRNPF for AS forward | AAGTGGTGGGATTACAGGCG |
| HNRNPF for AS reverse | ATCCGTGACTGCTCCATTGC |
| HNRNPF for DIP forward | CTTTGCCGGAAGCCCTCCT |
| HNRNPF for DIP reverse | CTCCTTGGGCTTCTGTTTCCT |
| PTBP1 for AS forward | TCGAACTGTACGAGCTCCA |
| PTBP1 for AS reverse | GGCTCCATACAATCCAAGG |
| PTBP1 for DIP forward | GTTCCTGCTATTCCGGCGC |
| PTBP1for DIP reverse | CGACGTGCGACTCACCCGT |
| IRS2  AS and DIP forward | AAGCAGATCTTGCAGCCCA |
| IRS2 AS and DIP reverse | CGTTGAGGTAGTCCCCGTT |
| CBS AS and DIP forward | ATGTATCCGTCCAGGTGAGAT |
| CBS AS and DIP reverse | GGTCAAGACCGCAGGCGTG |
| CIORF216 AS forward | GTCCCCGTCCCTAGCTTGCT |
| CIORF216 AS reverse | CAGGTTATGGAGGAGAGAAGAG |
| CIORF216 DIP forward | CGACACGCCAGGACTAGGT |
| CIORF216 DIP reverse | CTACGCCGTGAGTCCGAGT |
| PNP AS forward | CTGGGAAGACCAAACGTTGC |

| | |
|---|---|
| PNP AS reverse | ATGTCTGGTTTCCCGGGC |
| PNP DIP forward | GCAAGGGATATAAGCCAGAGC |
| PNP DIP reverse | CTTCTCGATGAGCCTGCTCC |

**Table S5: Primers for PIC validation (related to STAR Methods)**

| PRIMER'S NAME | PRIMER'S SEQUENCES (5'-3') |
|---|---|
| TRIM33 sense forward | ATGCCCAGCTTTCCCTAACT |
| TRIM33 sense reverse | GGAAAGTGGACTGCATGGTT |
| TRIM33 antisense forward | CCCTCGAAGCCAGTTCCTTG |
| TRIM33 antisense reverse | GGCTACCACCCTTCCGTATG |
| EHD1 sense forward | CTGAACATACTGCCGGACAC |
| EHD1 sense reverse | GACCCTATAAAGGCCGCTCC |
| EHD1 antisense forward | CATTGGCTGATTCCAAATCTC |
| EHD1 antisense reverse | TGACTGGGTGTCTCGTGGAG |
| NBPF1 sense forward | CTTCGCGTAACTTCCCATTC |
| NBPF1 sense reverse | ATCCTGAGGAGCCAGTGG |
| NBPF1 antisense forward | CGTGTCAGGATAACCCAAGG |
| NBPF1 antisense reverse | AGCGATTTGGAGGGTCGAG |
| SUCO sense forward | GGCTGTTGAGAGGCGAGTA |
| SUCO sense reverse | GAGAATGTGACCGATAGTCCTAAG |
| SUCO antisense forward | CCTTCCCCACTTTAGGTGAAC |
| SUCO antisense reverse | CCAAGAATCCTCTCATCTGATT |
| AAAS sense forward | GGCTAGATTCGTATGCGGAC |
| AAAS sense reverse | CGCAGGAAGAAGCTTTGGA |
| AAAS antisense forward | GCGGGCTCTCATAGCTACTG |
| AAAS antisense reverse | GTTTGCCGACTGCAGACGT |

**Table S6: siRNA used in this study (related to STAR Methods)**

| siRNAs | Source | Identifier |
| --- | --- | --- |
| siLuc (custom siRNA) | Sigma | Sequence (5'-3')<br>Sense: GAUUAUGUCCGGUUAUGUAUU<br>Antisense: [phos]UACAUAACCGGACAUAAUCUU |
| siTOP1 (human), ON-TARGETplus | Dharmacon (GE) | L-005278-00-0005 |
| siAQR (human), ON-TARGETplus | Dharmacon (GE) | L-022214-01-0005 |
| siSETX (human), ON-TARGETplus | Dharmacon (GE) | L021420-00-0005 |
