# Supplementary material for: R-Loops Promote Antisense Transcription across the Mammalian Genome
Source: Mol Cell. 2019 Nov 21;76(4):600–616.e6. doi: 10.1016/j.molcel.2019.10.002 (PMC6868509; doi:10.1016/j.molcel.2019.10.002)
Supplement: Document S2. Article plus Supplemental Information [file mmc2.pdf]

Molecular Cell

Article

R-Loops Promote Antisense Transcription across the Mammalian Genome

Graphical Abstract

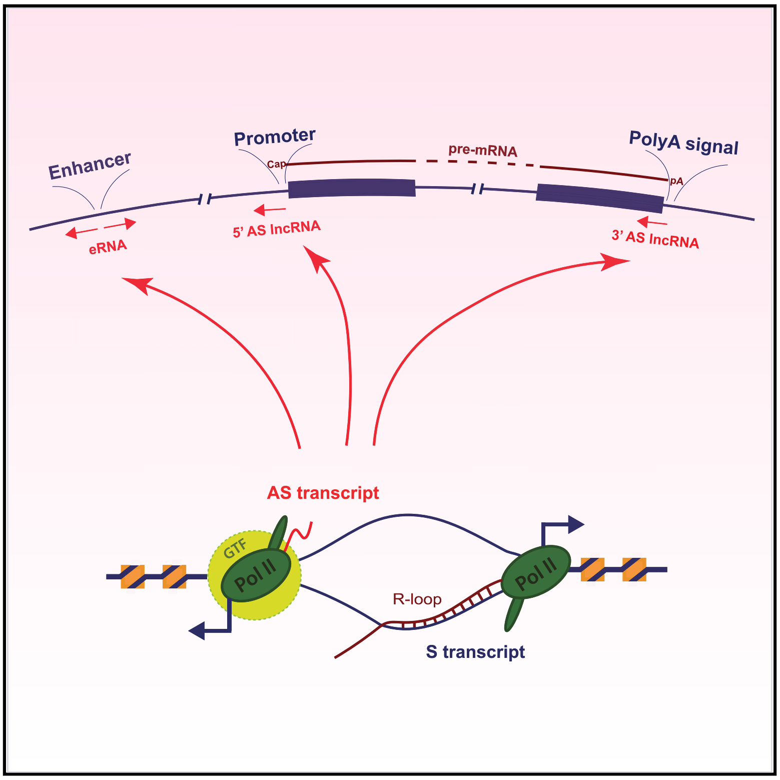

Highlights

- R-loops formed within plasmids promote antisense transcription in nuclear extracts
- TSS of lncRNA and eRNA are often near R-loop structures and sensitive to RNase H1
- Preinitiation complexes associated with lncRNA synthesis are R-loop dependent
- Many mammalian lncRNA derive from R-loop promoter activity

Authors

Sue Mei Tan-Wong, Somdutta Dhir, Nick J. Proudfoot

Correspondence

nicholas.proudfoot@path.ox.ac.uk

In Brief

Tan-Wong et al. demonstrate that R-loop structures, often formed at the promoter, terminator, and enhancer regions of human protein-coding genes, act as promoters to generate antisense lncRNA. In effect, R-loop promoter activity may account for the existence of many lncRNA that are detected across mammalian genomes.

Tan-Wong et al., 2019, Molecular Cell *76*, 600–616
November 21, 2019 © 2019 The Author(s). Published by Elsevier Inc.
https://doi.org/10.1016/j.molcel.2019.10.002

R-Loops Promote Antisense Transcription across the Mammalian Genome

Sue Mei Tan-Wong,[1,3] Somdutta Dhir,[1,2,3] and Nick J. Proudfoot[1,4,*]
[1]Sir William Dunn School of Pathology, University of Oxford, South Parks Road, Oxford OX1 3RE, UK
[2]Centre for Genomic and Experimental Medicine, IGMM, The University of Edinburgh, Western General Hospital, Crewe Road, Edinburgh EH42XU, UK
[3]These authors contributed equally
[4]Lead Contact
*Correspondence: nicholas.proudfoot@path.ox.ac.uk
https://doi.org/10.1016/j.molcel.2019.10.002

SUMMARY

Widespread antisense long noncoding RNA (lncRNA) overlap with many protein-coding genes in mammals and emanate from gene promoter, enhancer, and termination regions. However, their origin and biological purpose remain unclear. We show that these antisense lncRNA can be generated by R-loops that form when nascent transcript invades the DNA duplex behind elongating RNA polymerase II (Pol II). Biochemically, R-loops act as intrinsic Pol II promoters to induce *de novo* RNA synthesis. Furthermore, their removal across the human genome by RNase H1 overexpression causes the selective reduction of antisense transcription. Consequently, we predict that R-loops act to facilitate the synthesis of many gene proximal antisense lncRNA. Not only are R-loops widely associated with DNA damage and repair, but we now show that they have the capacity to promote *de novo* transcript synthesis that may have aided the evolution of gene regulation.

INTRODUCTION

A surprising feature of the mammalian genome is that only a small fraction directly encodes protein sequence. Thus, more than half of the genome comprises repetitive and normally inert sequence, while the remaining single-copy sequence is mainly noncoding, either intergenic or intronic in nature (Taft et al., 2007). This apparently low use of genomic sequence information for protein-coding capacity has in recent years been offset by surprising transcriptional complexity. In particular, almost all single-copy genomic DNA has the capacity to be transcribed at least in some cell types, even though only a minor fraction of these transcripts correspond to functional pre-mRNA sequence (Kapranov et al., 2007; Pelechano and Steinmetz, 2013; Schlackow et al., 2017). These extra long noncoding RNA (lncRNA) transcripts include long intergenic noncoding RNA (lincRNA) as well as enhancer RNA (eRNA) that initiate bi-directionally from numerous transcriptional enhancers that form a network of interactions with the promoters of protein-coding genes (Kim et al., 2010; Kowalczyk et al., 2012). Added to these separate lncRNA, most protein-coding genes themselves generate antisense (AS) lncRNA that initiate from their promoter (Jensen et al., 2013) or terminator (Skourti-Stathaki et al., 2014) regions, as well as internally from within intronic sequences (Mayer et al., 2015). It is the nature and origin of these AS transcripts that is the focus of our present study.

A further complexity of transcription is that nascent transcripts, as well as being subject to RNA processing, also have the capacity to anneal back to the DNA template strand. The formation of these RNA:DNA hybrids (hereafter called hybrids) with concomitant displacement of the coding strand as single-stranded DNA (ssDNA), called R-loop structures, is facilitated by the transcription process (Skourti-Stathaki and Proudfoot, 2014). Thus, elongating polymerase complexes transiently displace nucleosomes as well as under-winding (negative supercoiling) the DNA template, both of which favor R-loop formation. Although all transcription has the tendency to form R-loop structures, a range of activities act to restrict their formation. Either pre-mRNA packaging (Huertas and Aguilera, 2003) or rapid RNA processing such as splicing (Bonnet et al., 2017; Li and Manley, 2005) restricts R-loop formation by sequestering the newly formed transcript away from the DNA template. Even when formed, R-loops may be removed either by RNase H1 activity that selectively degrades RNA hybridized to DNA (Cerritelli and Crouch, 2009) or alternatively by various helicases such as Senataxin (SETX; Skourti-Stathaki et al., 2011), Aquarius (Sollier et al., 2014), DDX23 (Sridhara et al., 2017), and DHX9 (Cristini et al., 2018), which have all been shown to restrict R-loop accumulation. The likely reason for this anti-R-loop response is that if allowed to accumulate, R-loops can act as a major source of DNA damage either through the fragility of the exposed ssDNA or by acting as an impediment to DNA replication in the S phase of the cell cycle (Santos-Pereira and Aguilera, 2015). R-loops have also been closely associated with specific aspects of DNA rearrangement and repair and in these cases may be the feature of a particular functional lncRNA. This is true for both immunoglobulin heavy-chain class switching (Chaudhuri et al., 2003; Ribeiro de Almeida et al., 2018; Zheng et al., 2015) and chromosomal telomeric ends (Balk et al., 2013; Yu et al., 2014). Similarly, the repair of double-stranded DNA (dsDNA) breaks by homologous recombination is associated

 © 2019 The Author(s). Published by Elsevier Inc.
This is an open access article under the CC BY license (http://creativecommons.org/licenses/by/4.0/).

CellPress

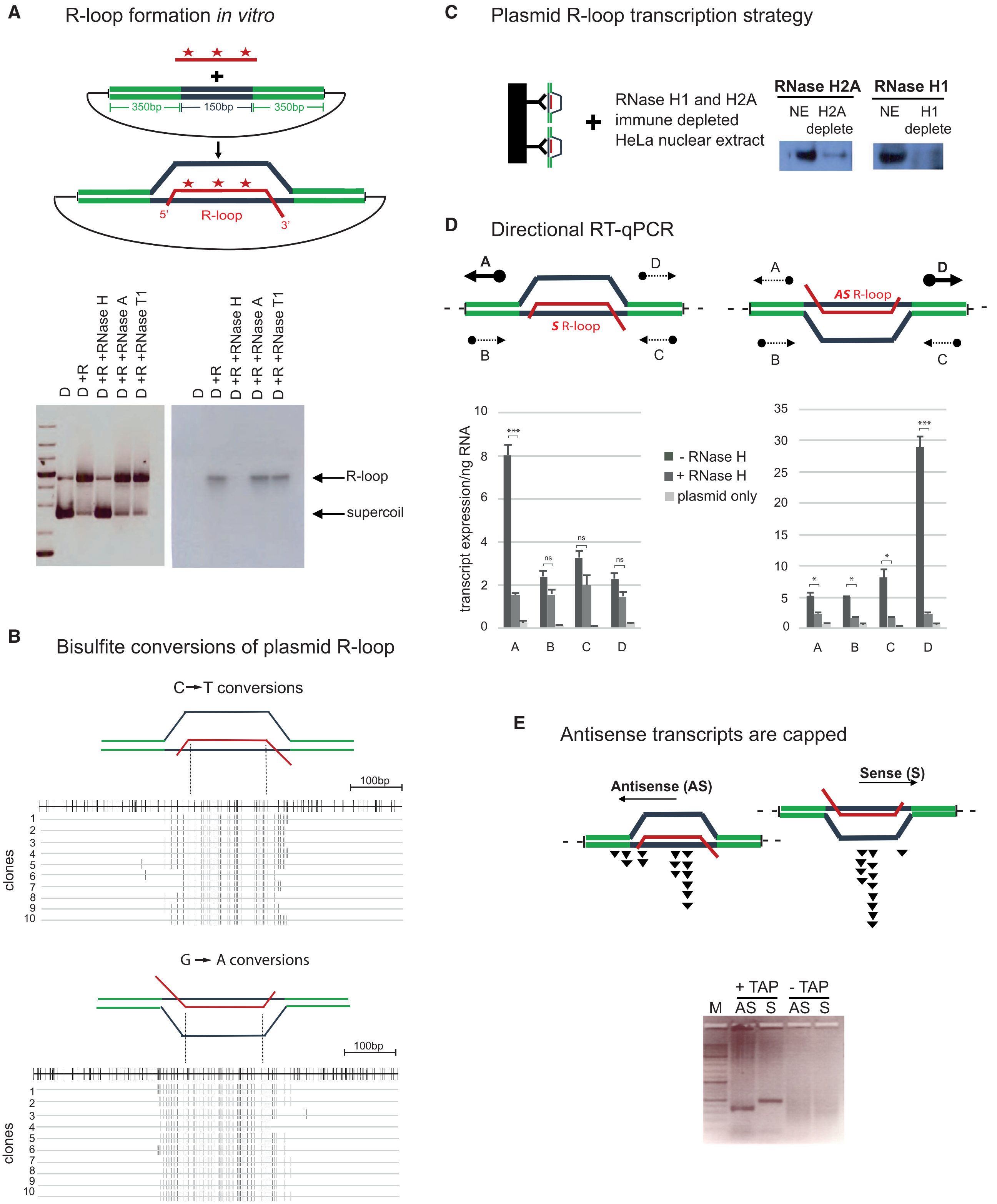

Figure 1. R-Loops Display *In Vitro* Promoter Activity

(A) R-loop plasmid construction: ^{32}P labeled (star) RNA (red line) was annealed to plasmid containing the G-rich β-actin gene 3′ end termination region (blue lines) flanked by *S. cerevisiae URA3* reporter gene (green lines). An ethidium bromide stained gel (left panel) and autoradiograph (right panel) show R-loops as slower migrating species, sensitive to RNase H but not A or T1 treatments. D, plasmid DNA; R, RNA.

(legend continued on next page)

with localized formation of R-loops that may act to facilitate the recruitment of DNA repair factors (D'Alessandro et al., 2018; Ohle et al., 2016).

It is well appreciated that RNA polymerase II (Pol II) initiates transcription more efficiently on accessible, nucleosome-depleted DNA templates, as found over gene promoters and terminators (Adelman and Lis, 2012; Grosso et al., 2012; Rhee and Pugh, 2012; Workman and Roeder, 1987). Similarly, the ssDNA component of R-loops has the potential to directly promote Pol II AS transcription, without the need for local double dsDNA unwinding by general transcription factors (GTFs). Thus early *in vitro* transcription experiments showed that supercoiled plasmids that possess underwound DNA duplex can initiate transcription with only Pol II and the minimal transcription factors TPB and TFIIB (Parvin and Sharp, 1993). It was also shown that DNA templates with specific heteroduplexed or mismatched promoter regions can initiate transcription using these same minimal transcription factors (Pan and Greenblatt, 1994). Both experiments suggest that a ssDNA template has the capacity to act as a Pol II promoter. We therefore predict that the ssDNA of the R-loop structure may similarly promote the selective expression of AS transcripts (opposite polarity to RNA in the hybrid). In this present study we demonstrate that artificially constructed R-loops can indeed act as *de novo* promoters in *in vitro* transcription experiments. We then extended these observations to *in vivo* transcription patterns in the human HeLa cell line. We demonstrate that the removal of R-loops selectively reduces AS transcription associated with many protein-coding genes and their transcriptional enhancers. Consequently, we show that R-loop formation facilitates the synthesis of a substantial fraction of lncRNA.

RESULTS

R-Loops Act as Promoters for *In Vitro* Transcription by Pol II

To test the possibility that R-loops possess intrinsic promoter activity, we generated an R-loop containing plasmid construct. The human β-actin gene terminator, which is prone to form R-loop structures (Skourti-Stathaki et al., 2014), was placed within *S. cerevisiae URA3* genic sequence, and this was then inserted into a circular plasmid. Both sense (S) and AS transcripts of the β-actin gene terminator were *in vitro* synthesized using bacteriophage RNA polymerase and annealed to the plasmid under conditions favoring R-loop formation. In an initial experiment, β-actin terminator RNA was α^{32}P-UTP labeled so that both DNA and radioactive RNA could be separately visualized by native agarose gel fractionation. R-loop containing plasmid migrated slower than supercoiled plasmid, as demonstrated by specific sensitivity to treatment with hybrid specific RNase H but not RNase A or T1 (Figure 1A). We elected to directly map the extent of the ssDNA formed by these plasmid-based R-loops. To this end, the R-loop plasmid preparation was treated with bisulfite, which demethylates dC to dT when in a ssDNA conformation. Individual plasmids were then isolated by bacterial transformation and cloning followed by the direct sequencing of ten selected plasmids. Notably C-T (or complementary G-A) mutations were detected directly over the R-loop region and extended by about 50 bp into the adjacent *URA3* sequence. From this analysis, it is evident that the ssDNA region of the R-loop plasmid expands beyond the hybrid region of 150 bp to a larger region of about 250 nt (Figure 1B).

R-loop-associated plasmids were then isolated using S9.6 antibody (specific for hybrid structures) immunoprecipitation (IP) and used as templates for *in vitro* transcription with HeLa cell nuclear extracts. Note that both the use of a circular DNA template and the fact that the annealed RNA contain small 3′ end non-complementary extensions prevented adventitious Pol II initiation on DNA or RNA 3′ ends. Also, nuclear extracts were first immuno-depleted of RNase H1 and H2A to avoid R-loop degradation of the template during the transcription reaction (Figure 1C). It is evident that this immunoselection procedure yielded R-loop plasmid preparations of at least 95% purity on the basis of their sensitivity to RNase H treatment (Figure S1A). Following *in vitro* transcription reactions using the S or AS R-loop plasmids, transcripts were mapped by qRT-PCR using four reverse transcriptase primers complementary to the flanking yeast *URA3* sequence (absent from the HeLa cell transcriptome). For both plasmids, a specific RNase H-sensitive signal was detected that appeared to initiate off the ssDNA formed by either the S or AS R-loop structures (Figure 1D). This result was confirmed by 5′ RACE (rapid amplification of cDNA ends), which revealed 5′ end capped (TAP-dependent) S and AS transcripts. Following their sequence analysis, a window of newly initiated transcripts was evident from within the ssDNA region of the R-loop (Figure 1E; Figure S1B). Overall, our *in vitro* transcription studies reveal that R-loops possess intrinsic promoter activity. Compared with adenovirus major late promoter (AdMLP), a well-characterized and efficient Pol II *in vitro* promoter (Parvin and Sharp, 1993), it is apparent that R-loop promoters display about 10% efficiency (Figure S1C). This raises the interesting and testable possibility that R-loops also facilitate initiation of transcription *in vivo*. Such an effect could account for the well-documented AS transcription associated with protein-coding genes (Kapranov et al., 2007; Pelechano and Steinmetz, 2013).

(B) PCR amplification and sequencing of ten cloned plasmids following bisulfite treatment of S or AS R-loop plasmid. The upper reference line (in black) depicts every potential C to T (upper panel) or G to A (lower panel) conversion. Gray lines show individual clones with C-to-T or G-to-A changes, respectively.

(C) Diagram showing S9.6 antibody immobilized on Dynabeads (black bar) and R-loop containing plasmids selected for transcription using HeLa nuclear extracts (NE). These were immuno-depleted for RNase H1 and H2A, as shown by western blot analysis.

(D) Diagram and quantitation of qRT-PCR analysis on *in vitro* transcribed RNA. Strand-specific RT primers were used to distinguish transcript orientation, as indicated by arrows (see Table S2). Thick arrows denote more abundant transcripts. Data are represented as means ± SEM (n = 3; *p < 0.05, **p < 0.01, and ***p < 0.001, paired t test).

(E) Gel fractionation of AS and S 5′ RACE products with or without tobacco acid pyrophosphatase (TAP) treatment. In upper diagram, black triangles denote RNA 5′ ends on the basis of sequence analysis (see Figure S1B).

Genome-wide R-Loop Profiles

The capacity of R-loops to act as intrinsic Pol II promoters, at least *in vitro*, led us to consider the possibility that R-loops generated *in vivo* might also facilitate synthesis of AS lncRNA. To establish a physiological role for R-loop promoter activity, we first needed to establish reliable R-loop profiles across the HeLa cell genome, our experimental human cell line.

Two main approaches have been used to characterize R-loop distribution across genomes. Initially, the hybrid specific antibody S9.6 (Boguslawski et al., 1986) was widely used, and it remains a "workhorse" for R-loop detection both in chromatin analysis and nuclear imaging. S9.6 signals that are sensitive to treatment by RNase H are widely taken to reflect the presence of R-loop structure. However, S9.6 specificity for hybrid nucleic acid is incomplete, as it also recognizes RNA duplex structures, albeit with reduced affinity (Hartono et al., 2018). Notably, other nucleic acid structures, including RNA G-quadruplex, do not significantly bind this antibody (Figure S2A). Several studies have described the distribution of R-loops across the human genome on the basis of the use of S9.6 IP of hybrid genomic nucleic acid (Ginno et al., 2012; Nadel et al., 2015). However, because of the lack of published, strand-specific R-loop profiles for HeLa cells, we generated our own genomic R-loop profile and obtained related data to that of the human embryonic carcinoma cell line Ntera2 (Sanz et al., 2016). In both studies, the RNA moiety of the R-loop is directly sequenced to give orientation-specific and higher resolution profiles. A comparison of our genome-wide R-loop profile (using RNA:DNA IP followed by cDNA sequencing [RDIP-seq]) for HeLa cells versus the Ntera2 profile (using DNA:RNA IP followed by cDNA sequencing [DRIPc-seq]) shows a partial overlap, with about 25% of the NTera2 R-loop peaks also present in the HeLa cell profile (Figure S2B). This low correspondence of R-loop peaks between the two databases reflects both the different patterns of expression between these two cell lines as well as technical differences between these two library preparation procedures. As shown for the *AARS2* gene (Figure S2C), the DRIPc-seq profile gives signal across this whole 13 kb gene, but with some transcription start site (TSS) and transcription end site (TES) accumulation. In contrast, the RDIP-seq profile is more specific, with the major signal over the gene TES region. Metagene analysis of 994 protein-coding genes from both cell types shows more defined TSS and TES peaks for RDIP-seq compared with DRIPc-seq profiles (Figure S2D). We also note that more than 90% of RDIP peaks are sensitive to RNase H1 overexpression, and nearly all displayed RNase H sensitivity following immunoselection. All these RDIP-seq libraries showed significant reproducibility (Figure S2E).

As a way to complement the detection of hybrid by S9.6, we also used mutated RNase H1 that binds but does not cleave hybrid structure (Chen et al., 2017). Transfection of expression plasmids for this mutated RNase H1, tagged with GFP, allows the application of a straightforward chromatin IP sequencing (ChIP-seq) protocol. Transfected, mutant RNase H1 (D210N) versus a negative control RNase H1 that lacks both hybrid binding and RNase H1 activity (WKKD) can be crosslinked to chromatin by formaldehyde treatment followed by IP of sonicated chromatin with Tag-specific antibody. Sequence analysis of the recovered DNA from these IPs yielded genome-wide R-loop profiles for the human cell line HEK293, referred to as the R-ChIP-seq method (Chen et al., 2017). Furthermore, because of the selective loss of the ssDNA in these isolated R-loops, the profile obtained appears relatively strand specific. We elected to modify R-ChIP-seq by overexpressing GFP-tagged D210N and WKKD RNase H1 in HeLa cells. These GFP-tagged RNase HI constructs were expressed at 4-fold higher levels following HeLa cell transfection than endogenous RNase H1. Also following IP of sonicated chromatin with GFP antibody, we isolated the RNA moiety from the hybrid by DNase treatment and used it as a template for Illumina RNA directional sequencing, a procedure we call RR-ChIP-seq (Figure 2A). Replicate RR-ChIP-seq libraries showed higher reproducibility than for RDIP-seq libraries (Figures S2E and S2F)

Initial scrutiny of two genes, *PRDX1* and *PNP*, co-expressed in both HeLa and HEK293 cells (Figure 2B) shows similar R-loop profiles with RR-ChIP-seq and R-ChIP-seq data, although the former profiles are fully strand specific and more tightly localized (see also *ATAD2* and *AARS2* R-loop profiles in Figures S2G and S2C). We next carried out metagene analysis of RR-ChIP-seq signals over TSS and TES regions of protein-coding genes (Figure 2C). Notably, the highest and most defined peaks (using D210N RNase H1) were detected over the TSS. Little signal was observed with the negative control WKKD RNase H1 profile over the TSS. Also, AS signal was low level and likely not significant. For the TES region, positive signal was obtained up to the TES region but at lower levels than for TSS R-loops and without a clear signal peak. Furthermore, wild-type RNase H1 overexpression (pRNH1-GFP) gave no signal above background over either the TSS or the TES region. Quantitation of R-loop signal for the different lncRNA categories compared with pre-mRNA in all cases showed R-loop signals above the negative control (WKKD) but at lower levels than seen with pre-mRNA transcripts (Figure 2D). We finally compared our RR-ChIP-seq profiles with those obtained using RDIP-seq (Figure S2H). Notably the RR-ChIP-seq signals were generally similar in size, but more TSS-centric. Thus, RDIP-seq profiles were more widespread across the whole protein-coding gene. We reason that the overexpression of the catalytically dead RNase H1 in HeLa cells may favor R-loop detection nearer TTS regions as its binding will be irreversible and so may select for earlier gene transcription. Instead, for RDIP-seq, S9.6 antibody is used *ex vivo* (following chromatin isolation) so that the R-loop profile may be less TSS biased. For this reason, both RR-ChIP-seq and RDIP-seq profiles were used in our subsequent analyses.

Many AS lncRNA Are RNase H1 Sensitive

We next analyzed genome-wide chromatin-associated RNA (ChrRNA-seq) from HeLa cells as a measure of mainly nascent transcription (Nojima et al., 2015). Furthermore, we obtained a second ChrRNA-seq profile from HeLa cells engineered to overexpress exogenous RNase H1 (Cerritelli et al., 2003) (Figure 2A). To obtain a homogeneous cell population of RNase H1 overexpression, HeLa cells transfected with either a plasmid expressing GFP-tagged RNase H1 or a GFP alone control plasmid were purified by fluorescence-activated cell sorting (FACS) (see STAR Methods). These two sorted HeLa cell populations

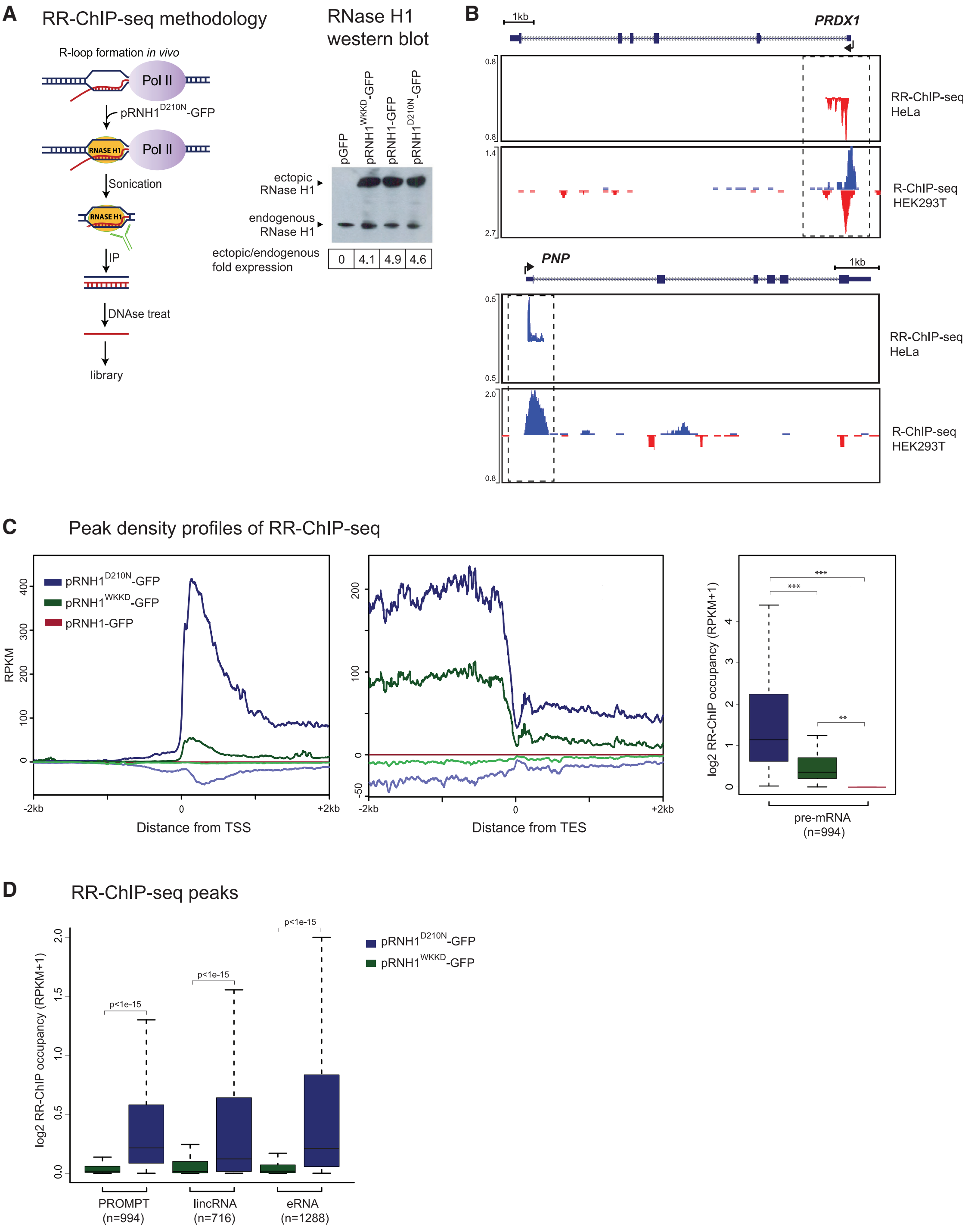

Figure 2. Genomic Analysis of R-Loop Distribution

(A) Schematic of RR-ChIP-seq methodology (left panel). Hybrid interaction with catalytic mutant D210N RNase H1 is used to create a directional RNA library. Right panel shows expression levels of GFP-tagged ectopic RNase H1 (pRNH1-GFP) and mutants by western blot. WKKD is a non-binding catalytic mutant (W43A, K59A, K60A, and D210N).

(B) RR-ChIP-seq and R-ChIP-seq peak profiles for *PRDX1* and *PNP*.

(C) Meta-analysis (left and middle panels) and boxplots (far right panel) of averaged RR-ChIP-seq signals of protein-coding genes across ±2 kb genomic region flanking TSS and TES regions for pRNH1 D210N and WKKD mutants and wild-type RNase H1 ($p = 1.5e-09$, Kruskal-Wallis test). Notably, almost all RR-ChIP-seq peaks disappear with the wild-type RNase H1 condition.

(D) Boxplots of RR-ChIP-seq signal over various genomic locations for D210N and WKKD mutants for PROMPTs (3 kb from TSS), pre-mRNA (whole annotated gene), lincRNA (TSS to TES), and eRNA (2 kb from center).

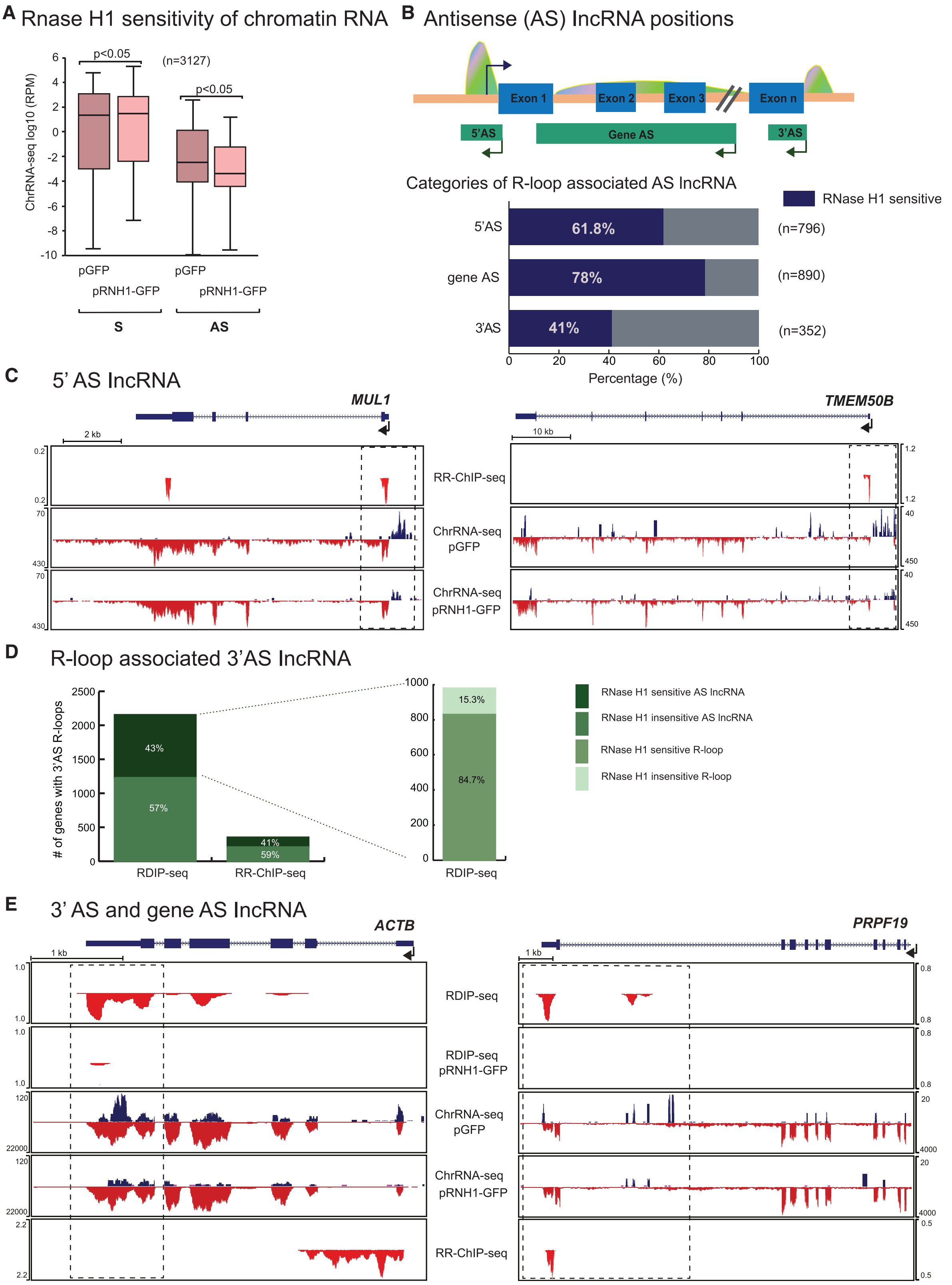

(legend on next page)

were then used in ChrRNA-seq library generation. Duplication of these ChrRNA-seq libraries shows consistent results (Figure S3A).

As an initial comparison of the HeLa cell transcriptome with or without RNase H1 overexpression, we note that overall S transcription from protein-coding gene TSS regions slightly increases, whereas AS transcription is significantly reduced following RNase H1 overexpression (Figure 3A). We next subdivided AS lncRNA derived from specific protein-coding genes into three categories: promoter-associated or 5′AS, intragenic or gene AS, and terminator-associated or 3′AS. On the basis of stringent algorithms, we identified numerous AS lncRNA peaks in all three categories that were within 0.5 kb of adjacent R-loop peaks and that showed 1.5-fold or greater reduction in levels following RNase H1 overexpression (Figure 3B). We also present pie diagrams showing the total number of R-loop peaks (on the basis of RR-ChIP-seq) corresponding to 5′AS, gene AS, and 3′AS transcripts and the fraction of these lncRNA that display RNase H sensitivity (Figure S3B). It is evident from all the bioinformatic data presented (Figures 3A, 3B, and S3B) that a substantial number of R-loop peaks are associated with RNase H1-sensitive AS lncRNA genome-wide. In particular for the 5′AS lncRNA, 73.8% of the RNase H1-sensitive fraction also corresponds to RNase H1-sensitive R-loops (Figure S3B, lower panel). Specific examples of R-loop-associated 5′AS lncRNA are shown for *MUL1* and *TMEM50B* (Figure 3C). In both cases, the pre-mRNA detected by ChrRNA-seq shows predominantly exonic reads reflecting co-transcriptional splicing (Nojima et al., 2018a), but these profiles are largely unaffected by RNase H1 overexpression. In contrast, lower level 5′AS lncRNA appear slightly shifted from the TSS-associated R-loops so that they are in a position indicative of R-loop promoter activity. Consistent with this scenario, both these 5′AS lncRNA are greatly reduced in level following RNase H1 overexpression. Note that sporadic AS reads are also detected across *TMEM50B*, which are also largely RNase H1 sensitive. These may be associated with additional R-loops below detection levels in the RR-ChIP-seq profile.

As described above (Figure S2H), we detect some TSS-associated bias for R-loop peaks detected by RR-ChIP-seq methodology. In contrast the RDIP-seq procedure appears to reveal additional R-loop peaks further into transcription units. Indeed, bioinformatic analysis of TES proximal R-loop peaks showed 5 times more R-loop peaks from our RDIP-seq than RR-ChIP-seq libraries. However, a similar ~40% proportion of defined R-loop peaks correlated with RNase H1-sensitive lncRNA. Notably almost 85% of these particular R-loop peaks were also RNase H1 sensitive (Figure 3D). Finally, specific examples of 3′AS lncRNA for *ACTB* and *PRPF19* are shown (Figure 3E). For *ACTB*, RR-ChIP-seq gives TSS, while RDIP-seq gives more TES-centric R-loop profiles. Low-level 3′AS lncRNA is evident over *ACTB* that shows RNase H1 sensitivity. These profiles are consistent with previous gene specific analysis of *ACTB* 3′-associated R-loops and 3′AS lncRNA (Skourti-Stathaki et al., 2011, 2014). For *PRPF19*, 3′-positioned R-loop peaks are evident in both the RR-ChIP-seq and RDIP-seq profiles, and again 3′AS or gene AS lncRNA show clear RNase H1 sensitivity, indicative of R-loop promoter activity. Note that for both *ACTB* and *PRPF19*, the RDIP-seq peaks are both sensitive to RNase H1 overexpression. Overall, we conclude that AS lncRNA often correlate with R-loop peaks. Notably, their clear sensitivity to RNase HI overexpression implies widespread R-loop-dependent promoter activity.

We have recently shown that loss of the elongation factor SPT6 not only reduces protein-coding gene transcription but also results in elevated levels of lncRNA. Notably these induced lncRNA are prone to form R-loop structures (Nojima et al., 2018b). We therefore looked for cases in which R-loop signals are detectable on both DNA strands, formed by either pre-mRNA or adjacent lncRNA (Figure S3C). Only 10% of AS lncRNA form detectable R-loops on the basis of our RR-ChIP-seq data. However, these are notably more RNase H1 sensitive than the majority of AS lncRNA that do not form R-loops. Furthermore, pre-mRNA associated with AS lncRNA that directly form R-loops also show modest RNase H1 sensitivity. This contrasts with the bulk of pre-mRNA that are associated only with S R-loops. In this case pre-mRNA are slightly elevated in levels following RNase H1 overexpression (Figures 3A and S3C). Specific examples of a gene that displays only a S R-loop (*MCMBP*) or both a S and AS R-loop (*WAPL*) are presented to exemplify this double-R-loop phenomenon (Figure S3D)

eRNA Are Often R-Loop Associated

Previous studies have indicated that enhancer transcription can be associated with R-loop formation (Nojima et al., 2018b; Pefanis et al., 2015). We therefore systematically searched for R-loop association with eRNA formation over HeLa cell enhancers. Enhancer regions were selected that generated the highest eRNA levels from our HeLa cell ChrRNA-seq data. Of these 316 enhancer regions, 26% had R-loop peaks (on the basis of RR-ChIP-seq), and half of these showed RNase HI sensitivity (Figures 4A and 4B). Two specific examples of enhancers taken from different chromosomes show both orientations of R-loop signal focused on the center of the enhancer (boxed with dashed

Figure 3. RNase H1-Sensitive AS Transcripts

(A) Boxplots of ChrRNA-seq expression levels for 3,127 protein-coding genes in pGFP and pRNH1-GFP-positive cells. S and AS regions correspond to ±3 kb from TSS in the direction of transcription. p values were computed using the Wilcoxon test.

(B) Diagram showing the positions of AS transcripts and associated R-loop peaks across schematic protein-coding gene (upper panel). Arrows denote transcription direction. Lower panel shows the number of AS transcripts derived from specific protein-coding genes that are positioned in a window of ±250 bp around the RR-ChIP peaks. Percentage of AS lncRNA that display RNase H1 sensitivity is indicated.

(C) Specific examples of 5′AS lncRNA showing RNase H1 overexpression sensitivity in *MUL1* and *TMEM50B*.

(D) Stacked bar chart showing proportion of RNase H1 overexpression sensitive and insensitive transcripts overlapping with RDIP-seq or RR-ChIP-seq peaks within a region ±2 kb of annotated TES. Further breakdown (on the right) of RDIP-seq peaks overlapping RNase H1 overexpression sensitive transcripts that also show pRNH1-GFP sensitivity.

(E) *ACTB* and *PRPF19* show RNase H1 overexpression sensitivity in TES region.

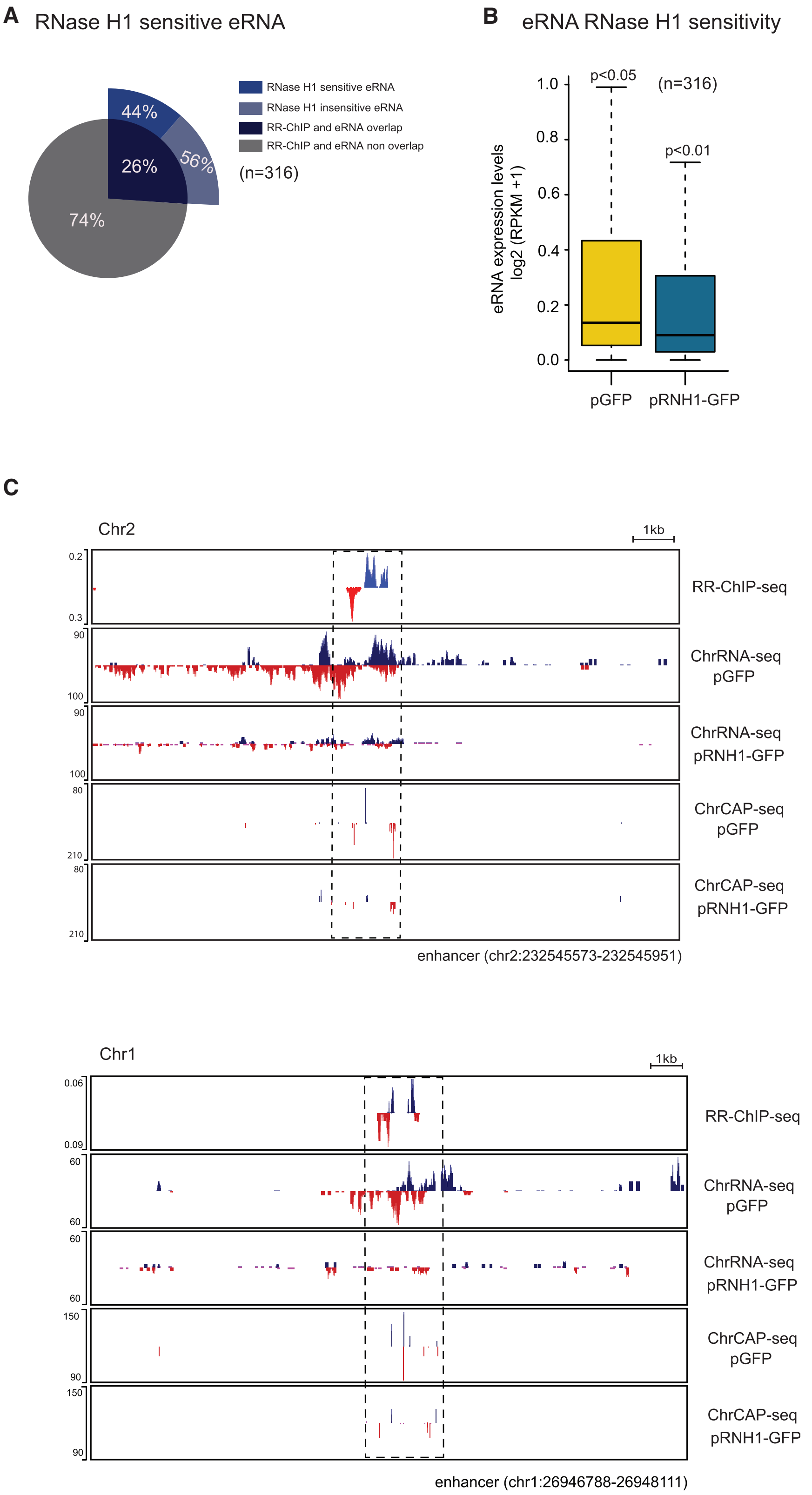

Figure 4. RNase H1-Sensitive eRNA

(A) Stacked pie chart showing distribution of all detectable eRNA overlapping with R-loops and sensitive to RNase H1 overexpression. Inner circle shows distribution of all expressed eRNA overlapping with R-loops, and outer ring shows frequency of RNase H1 overexpression sensitive eRNA.

(B) Boxplots comparing expression levels of eRNA with or without RNase H1 overexpression. p values were calculated using the Wilcoxon test.

(C) Two specific enhancers that display RNase H1-sensitive transcripts.

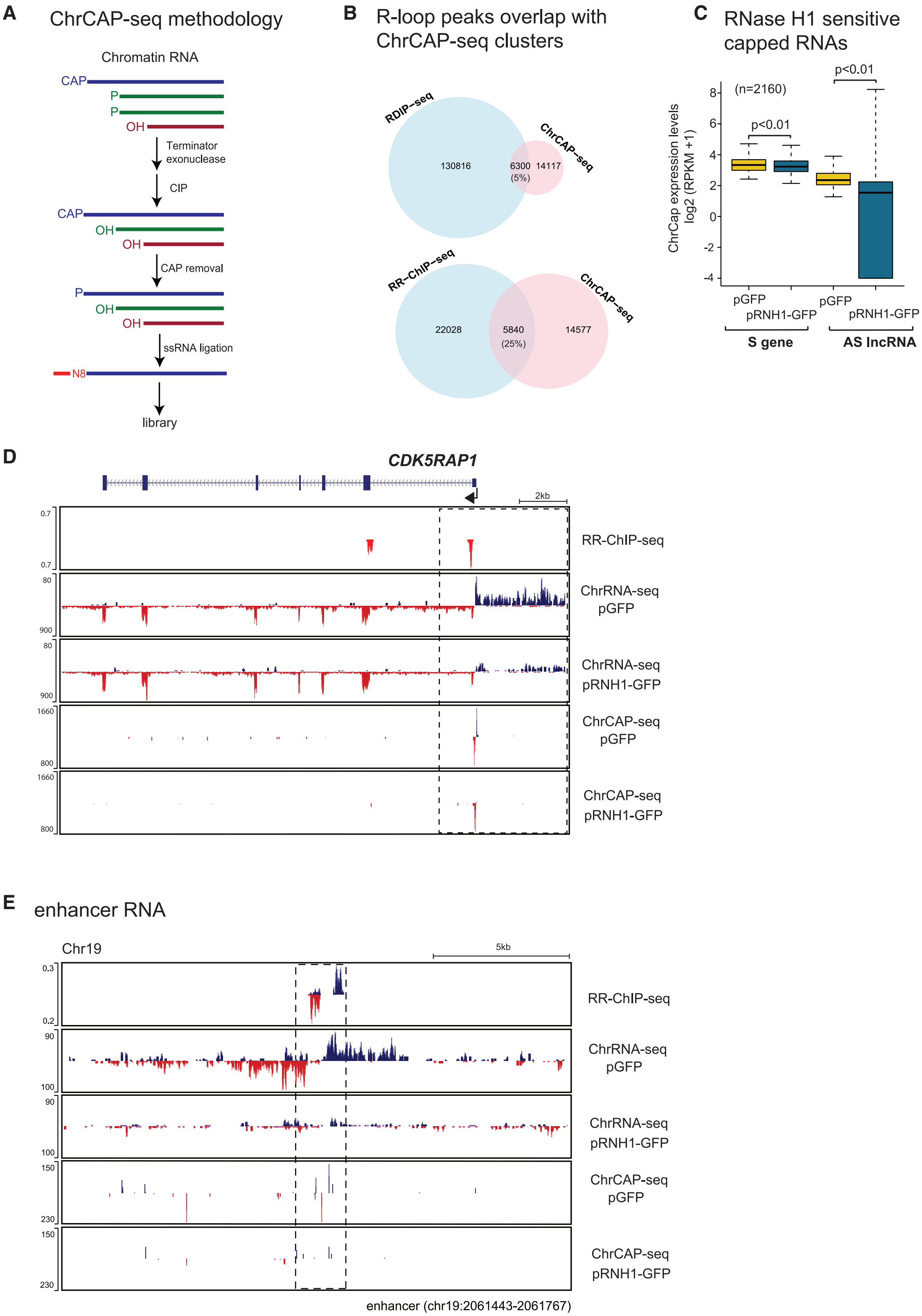

(legend on next page)

line) with eRNA reads spanning out from this central region (Figure 4C). Remarkably RNase H1 overexpression substantially reduced eRNA levels. Note that for both enhancers, some upstream reads are also detected, possibly reflecting transcripts that read into the enhancer region. Three further examples of eRNA associated enhancers are shown (Figure S4). In the chromosome X example, R-loops were detected in one orientation only, even though eRNAs were synthesized from both strands, and both showed clear RNase H1 sensitivity. Possibly the absence of R-loop signal on one strand may reflect differential R-loop stability. Also, because our analysis is on cell populations, it is possible that individual cells may generate predominantly unidirectional eRNA, as recently described (Kouno et al., 2019), and may also show orientation-specific RNase H1 sensitivity. Overall our analysis reveals that bidirectional transcripts (eRNA) associated with enhancers show clear evidence of R-loop promoter activity as a means to generate this widespread class of lncRNA.

Definition of TSS for *In Vivo* R-Loop Promoter Activity

The above results imply an association between AS lncRNA (including eRNA) and close-by R-loops, as many such transcripts display RNase HI sensitivity. However, we reasoned that clear evidence for R-loop promoter activity required the identification of RNase HI-sensitive TSS as defined by cap modification. To obtain such data, we modified existing Cap-seq technology as shown (Figure 5A). Essentially chromatin RNA is enzymatically digested with 5′P specific exonuclease (terminator) followed by phosphatase (CIP) to dephosphorylate the 5′ ends of all remaining RNA. Notably only 5′-capped TSS-associated RNA will be resistant to the above treatments. Following decapping, this RNA will retain a 5′P and so can be selectively tagged by a 5′ RNA linker using RNA ligase. Following standard library preparation, we obtained single-nucleotide resolution capped TSS profiles of chromatin RNA with or without RNase H1 overexpression. To first validate our ChrCAP-seq libraries, we compared them with existing 5′ GRO-seq (global run-on sequencing) libraries previously published for HeLa cells (Duttke et al., 2015). This latter TSS mapping procedure combines genomic nuclear run-on methodology with cap selection. Notably, of 994 protein-coding genes, identical TSS metagene profiles were obtained comparing these two methods (Figure S5A). Furthermore, a specific example of the convergent *BZW1-CLK1* locus shows closely similar TSS profiles for these two protein-coding genes (Figure S5B).

We next compared the association of TSS defined by our ChrCAP-seq data with R-loop peaks. Notably, the total number of R-loop peaks detected genome-wide by RR-ChIP-seq gave a 25% overlap with ChrCAP-seq peak clusters, while for the more numerous RDIP-seq detected R-loops, only 5% correspond to ChrCAP-seq peaks (Figure 5B). We therefore focused on RR-ChIP profiles for comparison with ChrCAP-seq defined TSS. We next compared the RNase H1 sensitivity of capped TSS for 2,160 protein-coding genes versus their associated AS lncRNA. Although the gene TSS were relatively insensitive to RNase H1 overexpression, the lncRNA TSS were much more sensitive. In particular, lncRNA TSS showed a wide range of sensitivities from complete to partial loss of signal (Figure 5C).

The above bioinformatic analysis is borne out by detailed scrutiny of individual gene examples (Figure 5D). Thus, for *CDK5RAP1*, the 5′AS lncRNA as shown by ChrRNA-seq is clearly RNase H1 sensitive and corresponds to an R-loop positioned over the gene TSS region. Remarkably, ChrCAP-seq peaks precisely define the TSS of both S and AS transcripts, but with the AS TSS showing selective and complete RNase H1 sensitivity. These data therefore establish that this AS lncRNA is indeed the product of R-loop promoter activity. We also compared ChrCAP-seq data with ChrRNA-seq data for an enhancer on chromosome 19 (Figure 5E) as well as the five different enhancers shown above (Figures 4C and S4). Clear ChrCAP-seq reads were detected for many of the eRNA, which were largely lost following RNase H1 overexpression. We note that ChrCAP-seq signals were often quite heterogeneous, possibly reflecting multiple TSS over enhancer regions.

Validation of *In Vivo* R-Loop Promoter Activity

We elected to evaluate and extend our ChrRNA-seq and ChrCAP-seq data by performing further analyses on three specific genes (*TRIM33*, *WHAMM*, and *LSM4*) that reveal clear R-loop-dependent AS lncRNA, two 5′AS and one 3′AS. As shown for *TRIMM33* and *WHAMM*, strong 5′AS lncRNA are evident, both substantially reduced following RNase H1 overexpression (Figures 6A and 6B). We note that the S TSS-proximal transcripts increase in levels following RNase H1 overexpression. This may reflect competition between these dual-orientation promoters. ChrCAP-seq matches these data by showing the loss or reduction in lncRNA capped transcripts, but not the protein-coding gene capped transcript, clearly indicative of R-loop promoter activity for both 5′AS lncRNA. These two cases provide further clear examples of 5′AS lncRNA driven by R-loop promote activity, as shown above (Figure 5D). We also present *LSM4* as an example of a 3′AS lncRNA generated by R-loop promoter activity (Figure 6C). Notably, this gene generates both R-loop-dependent 5′AS and 3′AS lncRNA. The R-loop profiles are quite complex over this gene, but R-loop peaks are detectable over the TSS on the basis of RR-ChIP-seq and TES detected by RDIP-seq. Note that the RDIP-seq-derived R-loop profile is sensitive to RNase H1 overexpression. The 5′AS lncRNA is clearly RNase HI sensitive on the basis of both

Figure 5. Cap Analysis of RNase H1-Sensitive Transcripts

(A) Schematic of ChrCAP-seq methodology.

(B) Overlap with CAP clusters of RDIP-seq peaks (top) and RR-ChIP-seq peaks (bottom).

(C) Boxplots showing expression level distribution for S and AS CAP signal for ChrCAP-seq clusters that overlap with AS transcript and are sensitive to RNase H1 overexpression. Statistical significance between distribution of medians assessed using the Wilcoxon test.

(D) *CDK5RAP1* displays RNase H1 capped sensitive 5’AS lncRNA.

(E) Chromosome 19 enhancer displays capped RNase H1-sensitive eRNA.

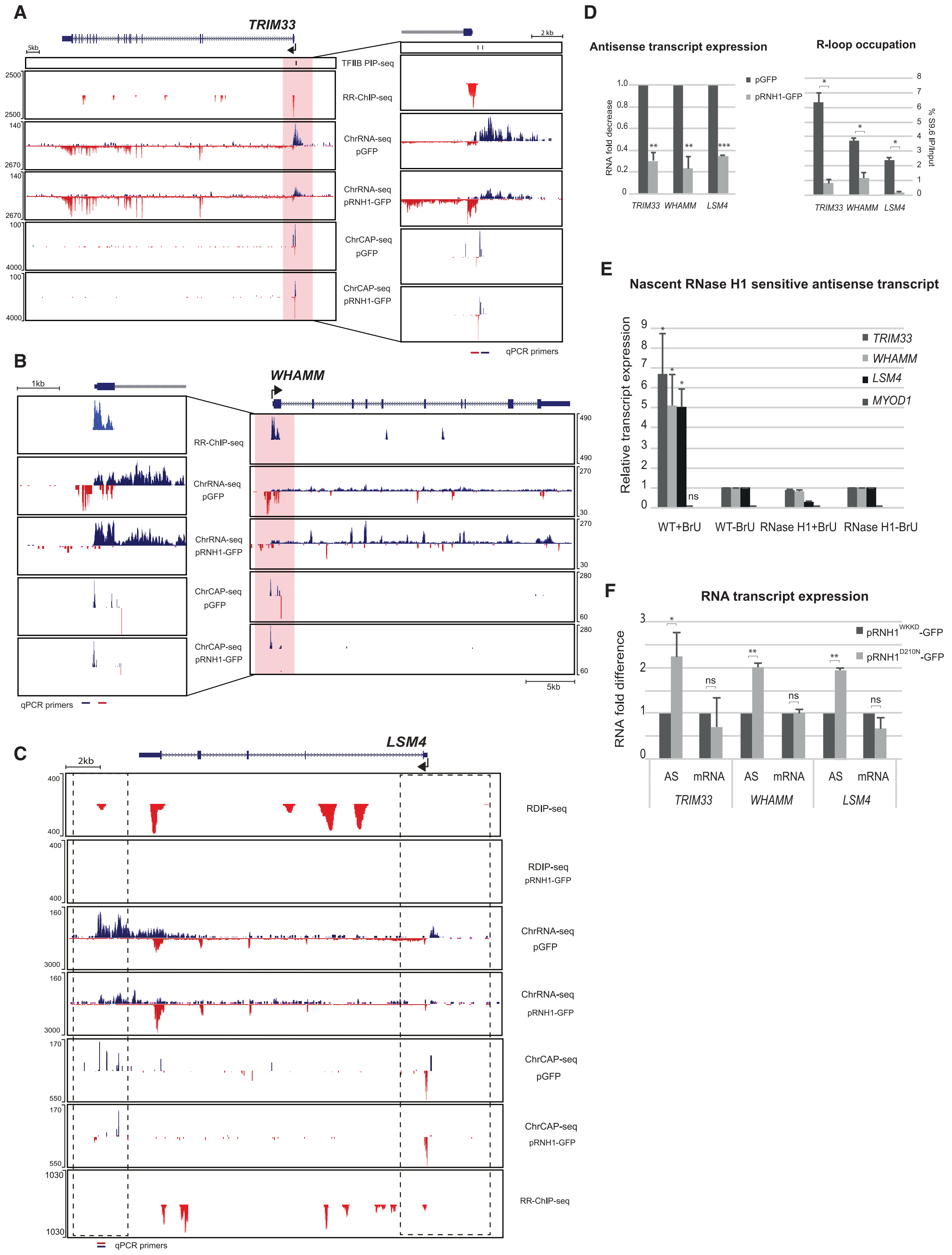

(legend on next page)

ChrRNA-seq and ChrCAP-seq. In contrast, the TES associated 3′AS lncRNA appears complex, with several separate RNase H1-sensitive capped 5′ ends evident.

To validate these three cases of R-loop-dependent lncRNA using specific qRT-PCR analysis, we first showed that each lncRNA is RNase H1 sensitive and associated with RNase H1-sensitive R-loops (Figure 6D). To extend this analysis, we selected a further ten genes that also display 5′AS lncRNA (Figure S6A). In all ten cases, the 5′AS lncRNA are again RNase H1 sensitive, each with a nearby positioned RNase H1-sensitive R-loop peak. We next carried out nuclear run-on analysis on the *TRIMM33*, *WHAMM*, and *LSM4* AS lncRNA promoters. Nuclei were isolated from HeLa cell transfected with either pGFP or pRNHI-GFP and subjected to *in vitro* transcription in the presence of Br-UTP. This modified nucleotide is incorporated into the run-on transcript, allowing its immunoselection (Skourti-Stathaki et al., 2011). Notably qRT-PCR of these run-on transcripts showed high levels of Br-U-labeled nascent RNA that is strongly sensitive to RNase H1 overexpression. The *MYOD1* promoter was used as a negative control, as it does not display any 5′AS lncRNA R-loop-dependent promoter activity (Figure 6E). These data provide independent evidence for the existence of R-loop promoter activity driving the synthesis of these three AS lncRNA. A further test for the requirement of an accessible R-loop structure to display promoter activity comes from the use of the mutant RNase H1 D210N. Remarkably, D210N mutant selectively enhances the synthesis of AS lncRNA compared with the non-binding RNase H1 control, WKKD (Figure 6F). Possibly the irreversible binding of D201N RNase H1 to R-loops over the RNA:DNA hybrid stabilizes the displaced DNA strand, so making it more effective as a *de novo* promoter of Pol II.

We finally investigated the effect of depleting factors that are known to restrict R-loop levels. Thus, loss of topoisomerase 1 (TOP1) enhances R-loop levels by preventing the removal of transcription associated negative supercoiling (Tuduri et al., 2009). Similarly, helicases SETX and Aquarius (AQR) have both been shown to remove transcription associated R-loops (Grunseich et al., 2018; Hatchi et al., 2015; Sollier et al., 2014). Small interfering RNA (siRNA) depletions of each of these three proteins (Figure S6B) causes a consistent, though variable activation level of R-loop-associated 5′AS lncRNA for the tested protein-coding genes. Overall our data establish, both by transcriptomic analyses and by different experimental manipulations, that R-loop-dependent AS lncRNA promoter activity is a widespread phenomenon across the HeLa cell genome.

AS lncRNA Transcription RNase H1-Sensitive PIC Formation

We reasoned that if R-loops can act as *de novo* promoters of AS transcription, then this effect should be associated with the formation of Pol II preinitiation complexes (PICs) of GTFs. We initially aligned a set of about 2,000 TSS-associated R-loop peaks as defined by our RR-ChIP-seq data with previously published promoter nuclease sensitivity and ChIP-exo data (Rhee and Pugh, 2012) for Pol II together with the GTFs, TBP, and TFIIB (Figure 7A). Heatmaps of these alignments clearly show that these TSS-associated R-loops correspond to nucleosome-depleted regions (NDRs). This confirms that R-loops may generally exclude nucleosomes. Furthermore, these R-loops are generally associated with double Pol II ChIP peaks, indicative of dual protein coding and PROMPT promoters. Similarly, TBP and TFIIB signals also accumulate over these R-loop peak regions, although resolution limitations prevent their separation into separate PICs for each promoter. To obtain a higher resolution PIC profile for these R-loop-associated TSS regions, we aligned recent H3K4me3 profiles obtained using mononucleosome DNA sequencing (mNuc-seq) (Nojima et al., 2018b). This technique involves the IP of H3K4me3-modified nucleosomes and sequencing-associated DNA. Two peaks of H3K4me3 indicative of separate promoters flanking the central NDR are evident (Figure 7A).

High-resolution PIC mapping has been carried out in human K562 cells using a genomic technique called protein interaction profile sequencing (PIP-seq) that co-isolates ssDNA chemically cleaved by permanganate and piperidine treatment formed at promoters with associated GTFs (Lai and Pugh, 2017). We therefore selected 5 Pol II promoters (for *TRMIM33*, *EHD1*, *NBPF1*, *SUCO*, and *AAAS*), which are highly expressed in both HeLa and K562 cells and all show separable PICs for S and AS transcription (Figures 6A and S7A–S7C). Note that the *AAAS* AS PIC is within exon 1, while for the four other genes the AS PIC is upstream of the S gene's PIC. For each of these dual-promoter regions, we carried out selective ChIP analysis with primer pairs over each S and AS PIC region using antibodies specific for a range of GTFs that together define the PIC: TFIIA, B, D-F, H, and TBP (Figures 7B and 7C). The ChIP signals obtained were normalized to input levels and then presented as a ratio of the signal obtained with or without RNase H1 overexpression. Remarkably, we mainly observed a reduction in signal for the AS but not S PICs. Although specific ChIPs show significant variation, there is a defined trend for lower ChIP signal over the AS PICs under conditions of RNase HI overexpression. Overall these preliminary results on the nature of R-loop promoter

Figure 6. Validation of *In Vivo* R-Loop Promoter Activity

(A–C) Comparisons of different R-loop and RNA profiles (RPM) for *TRIM33* (A), *WHAMM* (B), and *LSM4* (C) displaying RNase H1 capped sensitive AS lncRNA. Promoter regions for *TRIM33* and *WHAMM* (pink shading) shown in expanded view.

(D) Validation of AS lncRNA RNase H1 sensitivity by qRT-PCR with relative expression values normalized to 18S RNA (left). DIP analyses (Skourti-Stathaki et al., 2014) validate R-loop-occupied regions (right). Data are represented as mean ± SEM (n = 3; *p < 0.05, **p < 0.01, and ***p < 0.001, paired t test). qPCR primer locations are indicated (A–C) as red horizontal bars for R-loop location and blue for AS transcript expression.

(E) Bromo-UTP labeled nuclear run-on analysis of TSS R-loop promoter activity. Data are represented as mean ± SEM (n = 3; *p < 0.05, **p < 0.01, and ***p < 0.001, between WT+BrU and RNaseH1+BrU, paired t test).

(F) Quantification of S or AS transcripts by qRT-PCR, stabilizing R-loops with catalytic mutant pRNH1^{D210N}-GFP. Data are represented as mean ± SEM (n = 3; *p < 0.05, **p < 0.01, and ***p < 0.001, paired t test).

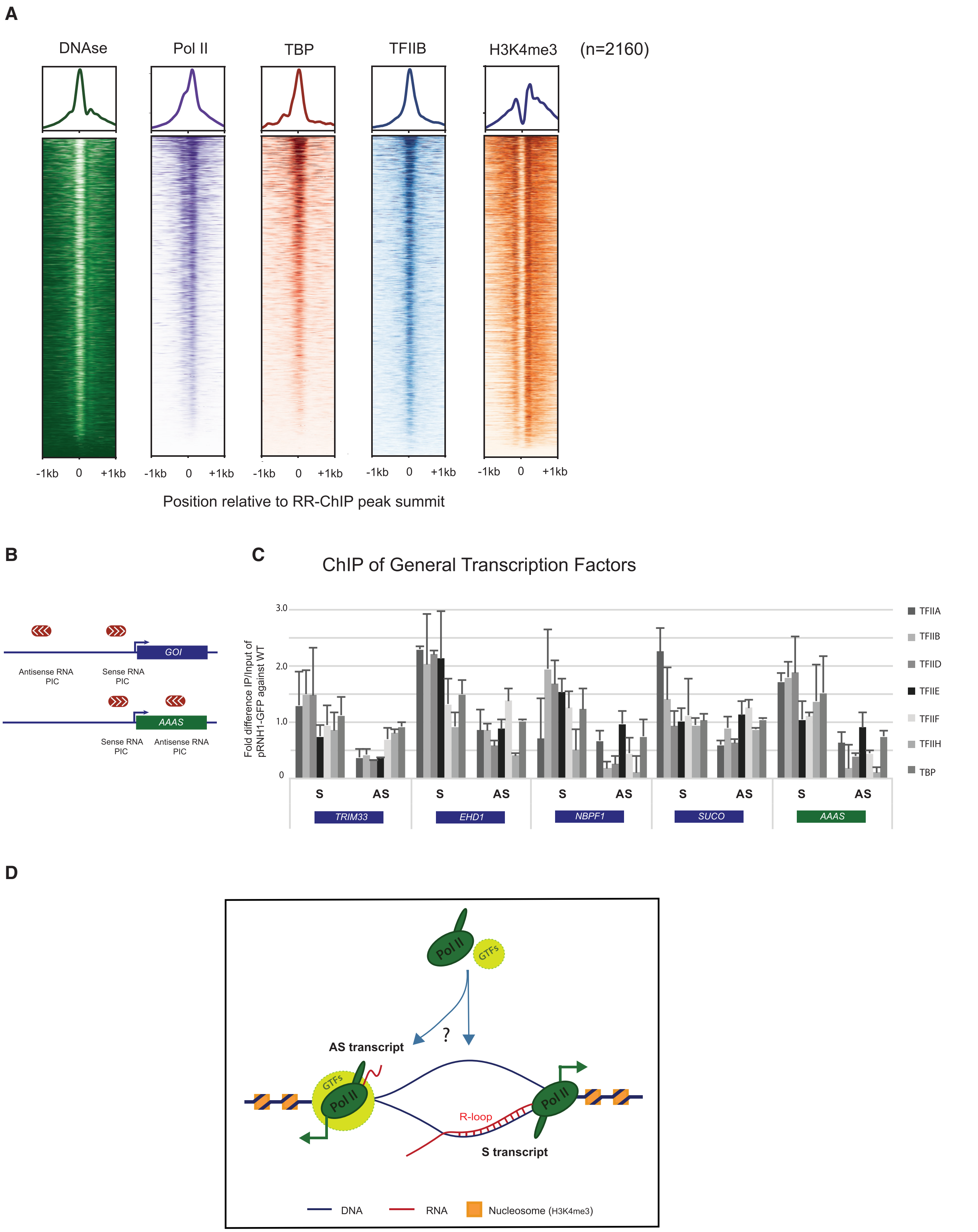

(legend on next page)

activity imply that these dynamic structures somehow influence the recruitment of PICs in an AS direction to the transcript that initially forms a hybrid with its template DNA strand (see model in Figure 7D). However, further biochemical and genetic analysis is required to better understand the molecular nature of this R-loop promoter activity. Even so, we establish here that R-loops promote the synthesis of AS lncRNA by acting as *de novo* promoters at numerous positions throughout the human genome.

DISCUSSION

We describe in this study the capacity of R-loops to act as inherent promoters for Pol II transcriptional initiation. Although our *in vitro* experiments (Figures 1 and S1) clearly show that R-loops have promoter capability, it has proved harder to define to what extent AS transcripts produced *in vivo* are truly R-loop dependent. In particular this is because the genome-wide analysis of R-loop profiles is limited by two aspects. First, only two R-loop copies can exist per nucleus for diploid loci. Consequently, R-loop signals will be potentially lower than transcript signals, which may reach much larger copy numbers per cell. Second, co-transcriptionally formed R-loops are known to be generated in competition with RNA packaging or processing activities. Consequently, the R-loops detected genome-wide are in effect steady-state profiles that remain in place throughout the genome, despite RNA packaging or processing as well as their direct removal by RNase H or hybrid helicase activities (Crossley et al., 2019; Santos-Pereira and Aguilera, 2015; Skourti-Stathaki and Proudfoot, 2014). Coupled with these theoretical issues, both existing methods for R-loop detection have technical limitations. First, the hybrid specific S9.6 antibody (used for RDIP-seq) also binds duplex RNA albeit with 3- to 4-fold lower efficiency (Figure S2A). Second, the mutated RNase H1 approach (RR-ChIP-seq) has limitations due to the possibility that its irreversible binding to R-loops may distort the physiological balance between transcription and R-loop formation. Even so, our genomic analysis, obtained using both methodologies (Figures 2 and S2), shows generally consistent profiles. This suggests that we are able to delineate many, although likely not all, R-loops across the HeLa cell genome.

Taken together, we establish in this study that a substantial fraction of AS RNA derived from protein-coding genes are R-loop associated. We note that our bioinformatic analyses have selected for stronger AS RNA peaks where there are sufficient reads to be certain of the transcript's existence. Also, as already mentioned, our genome-wide R-loop predictions may exclude less stable structures. Even so, we predict that many of the now well-documented AS RNA from transcribed protein-coding gene regions derive from intrinsic promoter activity of R-loops, as characterized in our *in vitro* transcription studies (Figure 1). We therefore demonstrate that R-loops have the clear capacity to define large parts of the mammalian transcriptome. Although many of these AS transcripts are likely rapidly degraded to prevent negative effects, some may have evolved into key regulators of gene expression. Added to these protein-coding gene-associated lncRNA, we also show that the potentially more numerous class of eRNA show clear R-loop promoter dependency. In effect, it is probable that a substantial fraction of lncRNA genome-wide owe their existence to R-loop-associated promoter activity.

Several previous studies are indicative of R-loop promoter activity. In the case of the vimentin gene, a TSS-proximal lncRNA that initiates in intron 1 was shown to enhance *VIM* transcript levels, possibly by setting up a more open chromatin environment over the *VIM* promoter (Boque-Sastre et al., 2015). Similarly, the *ZEB2* gene, which is associated with stem cell development and reprogramming, generates a lncRNA (referred to as a native AS RNA or NAT) initiating in the *ZEB2* intron 1 that plays a positive role in activating *ZEB2* expression. This is achieved in part by this AS lncRNA acting to block splicing of intron 1 to allow translation initiation on an IRES contained within this intron (Beltran et al., 2008). Recently, this lncRNA has also been shown to directly activate ZEB2 expression, suggesting a positive effect of this lncRNA on gene transcription (Bernardes de Jesus et al., 2018). Several previous examples of 3′AS lncRNA have also been described. These are associated with R-loops in the β-actin gene (*ACTB*) (Skourti-Stathaki et al., 2014) as well as several other pre-mRNA 3′ end regions (Neve et al., 2016), and may induce more efficient pre-mRNA 3′ end formation. Finally, an intriguing example of R-loop-associated promoter activity comes from a recent study on the role of transcription in double-strand break repair (Burger et al., 2019). Here transcripts that initiate on the 3′ ends of DNA breaks are shown to have a propensity to form R-loops, which in turn are associated with AS lncRNA, likely related to R-loop promoter activity.

It is worth considering the expanding impact R-loops can have on both genomic and transcriptomic integrity. It is now well established that R-loops can induce DNA damage by exposure of the displaced ssDNA to spontaneous or enzymatic mutation such as chemical oxidation or enzymatic deamination, respectively. It is also abundantly clear that the inherent stability of the hybrid structure can impede other copying enzymes, especially DNA polymerase. Because R-loops are associated with the stalling of associated RNA polymerase, this may in turn result in collision with oncoming replisomes, especially in head-on

Figure 7. Preinitiation Complexes on R-Loop Promoters

(A) Heatmap of RR-ChIP-seq signal density using k-means clustering showing correlation of RR-ChIP-seq peaks in a ±1 kb window with DNase-seq, Pol II, TBP, TFIIB ChIP-seq, and H3K4me3 mNuc-seq datasets.

(B) Schematic of PIC positions with respect to protein-coding gene TSS. GOI, gene of interest.

(C) qPCR quantification of ChIP signal for GTFs over regions as in (B). Data represented as mean ± SEM (n = 3).

(D) Model: Pol II and GTFs are recruited to R-loop regions through direct or indirect effects of ssDNA displaced by formation of RNA:DNA hybrid between S transcript and template DNA strand over the promoter. This will lead to synthesis of an antisense (AS) transcript through recruitment of an AS PIC either to the ssDNA or adjacent DNA. Positions of H3K4me3 nucleosomes that define bidirectional PICs are indicated.

orientation. Another major impact of R-loop structure is the provision of a sequence-specific recognition structure for binding of protein factors often with ssDNA binding capability (Cristini et al., 2018). Clear examples are the *Arabidopsis* protein AtNDX, which binds the ssDNA formed by a 3′AS lncRNA R-loop on *FLC*, the master regulator of plant vernalization. Recruitment of this factor plays a crucial role in the regulation of FLC (Sun et al., 2013). Also, recently the protein factor GADD45A has been shown to be recruited to the promoter of the tumor suppressor gene *TCF21*. This occurs through its interaction with an R-loop structure formed by the 5′AS lncRNA *TARID*. GADD45A so recruited then itself recruits the DNA demethylase TET1, which in turn will activate the TCF21 CpG island promoter by promoting DNA hypomethylation (Arab et al., 2019). It is further proposed that this may represent a general mechanism to activate a set of genes dependent on CpG island promoter demethylation. A range of other potential gene regulatory factors have been shown to associate with R-loops, suggesting that R-loops may provide a very general means of sequence-specific gene regulation. These examples of how R-loop structures may target gene-modifying factors clearly underlie their biological importance. Even so, we now show an additional layer of R-loop regulation whereby these structures act to expand the transcriptomic repertoire.

STAR★METHODS

Detailed methods are provided in the online version of this paper and include the following:

- KEY RESOURCES TABLE
- LEAD CONTACT AND MATERIALS AVAILABILITY
- EXPERIMENTAL MODEL AND SUBJECT DETAILS
- METHOD DETAILS
  - *In vitro* R-loop formation and transcription assay
  - Bisulfite conversions on *in vitro* plasmid R-loop
  - Cell transfection of siRNA and plasmids
  - Total RNA and chromatin-associated RNA (ChrRNA) library preparation
  - RNA quantitation by RT-qPCR
  - Br-UTP nuclear run-on analysis
  - Western blot
  - S9.6 validation by dot blot western
  - 5′ RACE
  - RNA specific RR-ChIP-seq
  - DIP and RNA specific RNA-DNA hybrid immunoprecipitation analysis (RDIP-seq)
  - Chromatin CAP-seq (ChrCAP-seq)
  - Chromatin Immunoprecipitation (ChIP) of GTFs
- QUANTIFICATION AND STATISTICAL ANALYSIS
  - Bioinformatic analysis
  - Data processing and visualization
- DATA AND CODE AVAILABILITY

SUPPLEMENTAL INFORMATION

Supplemental Information can be found online at https://doi.org/10.1016/j.molcel.2019.10.002.

ACKNOWLEDGMENTS

These studies were supported by a European Research Council (ERC) Advanced Grant (339270) and a Wellcome Trust Investigator Award (107928|Z|15|Z) to N.J.P. We are grateful to the N.J.P. lab for advice and discussion.

AUTHOR CONTRIBUTIONS

S.M.T.-W. preformed all the molecular biology and generated the genomic RNA-DIP and chromatin-seq databases. S.D. carried out all the bioinformatic analyses. N.J.P. and S.M.T.-W. designed the project and wrote the paper.

DECLARATION OF INTERESTS

The authors declare no competing interests.

Received: March 15, 2019
Revised: August 14, 2019
Accepted: September 30, 2019
Published: October 31, 2019

REFERENCES

Adelman, K., and Lis, J.T. (2012). Promoter-proximal pausing of RNA polymerase II: emerging roles in metazoans. Nat. Rev. Genet. *13*, 720–731.

Arab, K., Karaulanov, E., Musheev, M., Trnka, P., Schafer, A., Grummt, I., and Niehrs, C. (2019). GADD45A binds R-loops and recruits TET1 to CpG island promoters. Nat Genet *51*, 217–223.

Balk, B., Maicher, A., Dees, M., Klermund, J., Luke-Glaser, S., Bender, K., and Luke, B. (2013). Telomeric RNA-DNA hybrids affect telomere-length dynamics and senescence. Nat. Struct. Mol. Biol. *20*, 1199–1205.

Beltran, M., Puig, I., Peña, C., García, J.M., Alvarez, A.B., Peña, R., Bonilla, F., and de Herreros, A.G. (2008). A natural antisense transcript regulates Zeb2/Sip1 gene expression during Snail1-induced epithelial-mesenchymal transition. Genes Dev. *22*, 756–769.

Bernardes de Jesus, B., Marinho, S.P., Barros, S., Sousa-Franco, A., Alves-Vale, C., Carvalho, T., and Carmo-Fonseca, M. (2018). Silencing of the lncRNA Zeb2-NAT facilitates reprogramming of aged fibroblasts and safeguards stem cell pluripotency. Nat. Commun. *9*, 94.

Boguslawski, S.J., Smith, D.E., Michalak, M.A., Mickelson, K.E., Yehle, C.O., Patterson, W.L., and Carrico, R.J. (1986). Characterization of monoclonal antibody to DNA.RNA and its application to immunodetection of hybrids. J. Immunol. Methods *89*, 123–130.

Bonnet, A., Grosso, A.R., Elkaoutari, A., Coleno, E., Presle, A., Sridhara, S.C., Janbon, G., Geli, V., de Almeida, S.F., and Palancade, B. (2017). Introns protect eukaryotic genomes from transcription-associated genetic instability. Mol. Cell *67*, 608–621.e6.

Boque-Sastre, R., Soler, M., Oliveira-Mateos, C., Portela, A., Moutinho, C., Sayols, S., Villanueva, A., Esteller, M., and Guil, S. (2015). Head-to-head antisense transcription and R-loop formation promotes transcriptional activation. Proc. Natl. Acad. Sci. U S A *112*, 5785–5790.

Burger, K., Schlackow, M., and Gullerova, M. (2019). Tyrosine kinase c-Abl couples RNA polymerase II transcription to DNA double-strand breaks. Nucleic Acids Res. *23*, 3467–3484.

Cerritelli, S.M., and Crouch, R.J. (2009). Ribonuclease H: the enzymes in eukaryotes. FEBS J. *276*, 1494–1505.

Cerritelli, S.M., Frolova, E.G., Feng, C., Grinberg, A., Love, P.E., and Crouch, R.J. (2003). Failure to produce mitochondrial DNA results in embryonic lethality in Rnaseh1 null mice. Mol. Cell *11*, 807–815.

Chaudhuri, J., Tian, M., Khuong, C., Chua, K., Pinaud, E., and Alt, F.W. (2003). Transcription-targeted DNA deamination by the AID antibody diversification enzyme. Nature *422*, 726–730.

Chen, L., Chen, J.Y., Zhang, X., Gu, Y., Xiao, R., Shao, C., Tang, P., Qian, H., Luo, D., Li, H., et al. (2017). R-ChIP using inactive RNase H reveals dynamic

coupling of R-loops with transcriptional pausing at gene promoters. Mol. Cell *68*, 745–757.e5.

Cristini, A., Groh, M., Kristiansen, M.S., and Gromak, N. (2018). RNA/DNA hybrid interactome identifies DXH9 as a molecular player in transcriptional termination and R-Loop-associated DNA damage. Cell Rep. *23*, 1891–1905.

Crossley, M.P., Bocek, M., and Cimprich, K.A. (2019). R-loops as cellular regulators and genomic threats. Mol. Cell *73*, 398–411.

D'Alessandro, G., Whelan, D.R., Howard, S.M., Vitelli, V., Renaudin, X., Adamowicz, M., Iannelli, F., Jones-Weinert, C.W., Lee, M., Matti, V., et al. (2018). BRCA2 controls DNA:RNA hybrid level at DSBs by mediating RNase H2 recruitment. Nat. Commun. *9*, 5376.

Duttke, S.H.C., Lacadie, S.A., Ibrahim, M.M., Glass, C.K., Corcoran, D.L., Benner, C., Heinz, S., Kadonaga, J.T., and Ohler, U. (2015). Human promoters are intrinsically directional. Mol. Cell *57*, 674–684.

Flicek, P., Amode, M.R., Barrell, D., Beal, K., Billis, K., Brent, S., Carvalho-Silva, D., Clapham, P., Coates, G., Fitzgerald, S., et al. (2014). Ensembl 2014. Nucleic Acids Res. *42*, D749–D755.

Ginno, P.A., Lott, P.L., Christensen, H.C., Korf, I., and Chédin, F. (2012). R-loop formation is a distinctive characteristic of unmethylated human CpG island promoters. Mol. Cell *45*, 814–825.

Gromak, N., Dienstbier, M., Macias, S., Plass, M., Eyras, E., Cáceres, J.F., and Proudfoot, N.J. (2013). Drosha regulates gene expression independently of RNA cleavage function. Cell Rep. *5*, 1499–1510.

Grosso, A.R., de Almeida, S.F., Braga, J., and Carmo-Fonseca, M. (2012). Dynamic transitions in RNA polymerase II density profiles during transcription termination. Genome Res. *22*, 1447–1456.

Grunseich, C., Wang, I.X., Watts, J.A., Burdick, J.T., Guber, R.D., Zhu, Z., Bruzel, A., Lanman, T., Chen, K., Schindler, A.B., et al. (2018). Senataxin mutation reveals how R-loops promote transcription by blocking DNA methylation at gene promoters. Mol. Cell *69*, 426–437.e7.

Hartono, S.R., Malapert, A., Legros, P., Bernard, P., Chédin, F., and Vanoosthuyse, V. (2018). The affinity of the S9.6 antibody for double-stranded RNAs impacts the accurate mapping of R-loops in fission yeast. J. Mol. Biol. *430*, 272–284.

Hatchi, E., Skourti-Stathaki, K., Ventz, S., Pinello, L., Yen, A., Kamieniarz-Gdula, K., Dimitrov, S., Pathania, S., McKinney, K.M., Eaton, M.L., et al. (2015). BRCA1 recruitment to transcriptional pause sites is required for R-loop-driven DNA damage repair. Mol. Cell *57*, 636–647.

Huertas, P., and Aguilera, A. (2003). Cotranscriptionally formed DNA:RNA hybrids mediate transcription elongation impairment and transcription-associated recombination. Mol. Cell *12*, 711–721.

Hunter, J.D. (2007). Matplotlib: a 2D graphics environment. Comput. Sci. Eng. *9*, 90–95.

Jensen, T.H., Jacquier, A., and Libri, D. (2013). Dealing with pervasive transcription. Mol. Cell *52*, 473–484.

Kapranov, P., Willingham, A.T., and Gingeras, T.R. (2007). Genome-wide transcription and the implications for genomic organization. Nat. Rev. Genet. *8*, 413–423.

Kent, W.J., Sugnet, C.W., Furey, T.S., Roskin, K.M., Pringle, T.H., Zahler, A.M., and Haussler, D. (2002). The human genome browser at UCSC. Genome Res. *12*, 996–1006.

Kim, T.K., Hemberg, M., Gray, J.M., Costa, A.M., Bear, D.M., Wu, J., Harmin, D.A., Laptewicz, M., Barbara-Haley, K., Kuersten, S., et al. (2010). Widespread transcription at neuronal activity-regulated enhancers. Nature *465*, 182–187.

Kim, D., Pertea, G., Trapnell, C., Pimentel, H., Kelley, R., and Salzberg, S.L. (2013). TopHat2: accurate alignment of transcriptomes in the presence of insertions, deletions and gene fusions. Genome Biol. *14*, R36.

Kouno, T., Moody, J., Kwon, A.T., Shibayama, Y., Kato, S., Huang, Y., Böttcher, M., Motakis, E., Mendez, M., Severin, J., et al. (2019). C1 CAGE detects transcription start sites and enhancer activity at single-cell resolution. Nat. Commun. *10*, 360.

Kowalczyk, M.S., Hughes, J.R., Garrick, D., Lynch, M.D., Sharpe, J.A., Sloane-Stanley, J.A., McGowan, S.J., De Gobbi, M., Hosseini, M., Vernimmen, D., et al. (2012). Intragenic enhancers act as alternative promoters. Mol. Cell *45*, 447–458.

Lai, W.K., and Pugh, B.F. (2017). Genome-wide uniformity of human 'open' pre-initiation complexes. Genome Res. *27*, 15–26.

Langmead, B., and Salzberg, S.L. (2012). Fast gapped-read alignment with Bowtie 2. Nat. Methods *9*, 357–359.

Lee, D.Y., and Clayton, D.A. (1996). Properties of a primer RNA-DNA hybrid at the mouse mitochondrial DNA leading-strand origin of replication. J. Biol. Chem. *271*, 24262–24269.

Li, X., and Manley, J.L. (2005). Inactivation of the SR protein splicing factor ASF/SF2 results in genomic instability. Cell *122*, 365–378.

Li, H., Handsaker, B., Wysoker, A., Fennell, T., Ruan, J., Homer, N., Marth, G., Abecasis, G., and Durbin, R.; 1000 Genome Project Data Processing Subgroup (2009). The Sequence Alignment/Map format and SAMtools. Bioinformatics *25*, 2078–2079.

Liang, K., Woodfin, A.R., Slaughter, B.D., Unruh, J.R., Box, A.C., Rickels, R.A., Gao, X., Haug, J.S., Jaspersen, S.L., and Shilatifard, A. (2015). Mitotic transcriptional activation: clearance of actively engaged Pol II via transcriptional elongation control in mitosis. Mol. Cell *60*, 435–445.

Martin, M. (2011). Cutadapt removes adapter sequences from high-throughput sequencing reads. EMBnet.journal *17*, 10–12.

Mayer, A., di Iulio, J., Maleri, S., Eser, U., Vierstra, J., Reynolds, A., Sandstrom, R., Stamatoyannopoulos, J.A., and Churchman, L.S. (2015). Native elongating transcript sequencing reveals human transcriptional activity at nucleotide resolution. Cell *161*, 541–554.

Nadel, J., Athanasiadou, R., Lemetre, C., Wijetunga, N.A., Ó Broin, P., Sato, H., Zhang, Z., Jeddeloh, J., Montagna, C., Golden, A., et al. (2015). RNA:DNA hybrids in the human genome have distinctive nucleotide characteristics, chromatin composition, and transcriptional relationships. Epigenetics Chromatin *8*, 46.

Neve, J., Burger, K., Li, W., Hoque, M., Patel, R., Tian, B., Gullerova, M., and Furger, A. (2016). Subcellular RNA profiling links splicing and nuclear DICER1 to alternative cleavage and polyadenylation. Genome Res. *26*, 24–35.

Nojima, T., Gomes, T., Grosso, A.R.F., Kimura, H., Dye, M.J., Dhir, S., Carmo-Fonseca, M., and Proudfoot, N.J. (2015). Mammalian NET-seq reveals genome-wide nascent transcription coupled to RNA processing. Cell *161*, 526–540.

Nojima, T., Rebelo, K., Gomes, T., Grosso, A.R., Proudfoot, N.J., and Carmo-Fonseca, M. (2018a). RNA polymerase II phosphorylated on CTD serine 5 interacts with the spliceosome during co-transcriptional splicing. Mol. Cell *72*, 369–379.e4.

Nojima, T., Tellier, M., Foxwell, J., Ribeiro de Almeida, C., Tan-Wong, S.M., Dhir, S., Dujardin, G., Dhir, A., Murphy, S., and Proudfoot, N.J. (2018b). Deregulated expression of mammalian lncRNA through loss of SPT6 induces R-loop formation, replication stress, and cellular senescence. Mol. Cell *72*, 970–984.e7.

Ohle, C., Tesorero, R., Schermann, G., Dobrev, N., Sinning, I., and Fischer, T. (2016). Transient RNA-DNA hybrids are required for efficient double-strand break repair. Cell *167*, 1001–1013.e7.

Pan, G., and Greenblatt, J. (1994). Initiation of transcription by RNA polymerase II is limited by melting of the promoter DNA in the region immediately upstream of the initiation site. J. Biol. Chem. *269*, 30101–30104.

Parvin, J.D., and Sharp, P.A. (1993). DNA topology and a minimal set of basal factors for transcription by RNA polymerase II. Cell *73*, 533–540.

Pefanis, E., Wang, J., Rothschild, G., Lim, J., Kazadi, D., Sun, J., Federation, A., Chao, J., Elliott, O., Liu, Z.P., et al. (2015). RNA exosome-regulated long non-coding RNA transcription controls super-enhancer activity. Cell *161*, 774–789.

Pelechano, V., and Steinmetz, L.M. (2013). Gene regulation by antisense transcription. Nat. Rev. Genet. *14*, 880–893.

Pelechano, V., Wei, W., and Steinmetz, L.M. (2015). Widespread co-translational RNA decay reveals ribosome dynamics. Cell *161*, 1400–1412.

Quinlan, A.R., and Hall, I.M. (2010). BEDTools: a flexible suite of utilities for comparing genomic features. Bioinformatics *26*, 841–842.

Ramírez, F., Ryan, D.P., Grüning, B., Bhardwaj, V., Kilpert, F., Richter, A.S., Heyne, S., Dündar, F., and Manke, T. (2016). deepTools2: a next generation web server for deep-sequencing data analysis. Nucleic Acids Res. *44* (W1), W160–W165.

Rhee, H.S., and Pugh, B.F. (2012). Genome-wide structure and organization of eukaryotic pre-initiation complexes. Nature *483*, 295–301.

Ribeiro de Almeida, C., Dhir, S., Dhir, A., Moghaddam, A.E., Sattentau, Q., Meinhart, A., and Proudfoot, N.J. (2018). RNA helicase DDX1 converts RNA G-quadruplex structures into R-loops to promote IgH class switch recombination. Mol. Cell *70*, 650–662.e8.

Santos-Pereira, J.M., and Aguilera, A. (2015). R loops: new modulators of genome dynamics and function. Nat. Rev. Genet. *16*, 583–597.

Sanz, L.A., Hartono, S.R., Lim, Y.W., Steyaert, S., Rajpurkar, A., Ginno, P.A., Xu, X., and Chédin, F. (2016). Prevalent, dynamic, and conserved R-loop structures associate with specific epigenomic signatures in mammals. Mol. Cell *63*, 167–178.

Schlackow, M., Nojima, T., Gomes, T., Dhir, A., Carmo-Fonseca, M., and Proudfoot, N.J. (2017). Distinctive patterns of transcription and RNA processing for human lincRNAs. Mol. Cell *65*, 25–38.

Skourti-Stathaki, K., and Proudfoot, N.J. (2014). A double-edged sword: R loops as threats to genome integrity and powerful regulators of gene expression. Genes Dev. *28*, 1384–1396.

Skourti-Stathaki, K., Proudfoot, N.J., and Gromak, N. (2011). Human Senataxin resolves RNA/DNA hybrids formed at transcriptional pause sites to promote Xrn2-dependent termination. Mol. Cell *42*, 794–805.

Skourti-Stathaki, K., Kamieniarz-Gdula, K., and Proudfoot, N.J. (2014). R-loops induce repressive chromatin marks over mammalian gene terminators. Nature *516*, 436–439.

Sollier, J., Stork, C.T., García-Rubio, M.L., Paulsen, R.D., Aguilera, A., and Cimprich, K.A. (2014). Transcription-coupled nucleotide excision repair factors promote R-loop-induced genome instability. Mol. Cell *56*, 777–785.

Sridhara, S.C., Carvalho, S., Grosso, A.R., Gallego-Paez, L.M., Carmo-Fonseca, M., and de Almeida, S.F. (2017). Transcription dynamics prevent RNA-mediated genomic instability through SRPK2-dependent DDX23 phosphorylation. Cell Rep. *18*, 334–343.

Sun, Q., Csorba, T., Skourti-Stathaki, K., Proudfoot, N.J., and Dean, C. (2013). R-loop stabilization represses antisense transcription at the Arabidopsis FLC locus. Science *340*, 619–621.

Taft, R.J., Pheasant, M., and Mattick, J.S. (2007). The relationship between non-protein-coding DNA and eukaryotic complexity. BioEssays *29*, 288–299.

Trapnell, C., Williams, B.A., Pertea, G., Mortazavi, A., Kwan, G., van Baren, M.J., Salzberg, S.L., Wold, B.J., and Pachter, L. (2010). Transcript assembly and quantification by RNA-Seq reveals unannotated transcripts and isoform switching during cell differentiation. Nat. Biotechnol. *28*, 511–515.

Tuduri, S., Crabbé, L., Conti, C., Tourrière, H., Holtgreve-Grez, H., Jauch, A., Pantesco, V., De Vos, J., Thomas, A., Theillet, C., et al. (2009). Topoisomerase I suppresses genomic instability by preventing interference between replication and transcription. Nat. Cell Biol. *11*, 1315–1324.

West, S., Proudfoot, N.J., and Dye, M.J. (2008). Molecular dissection of mammalian RNA polymerase II transcriptional termination. Mol. Cell *29*, 600–610.

Workman, J.L., and Roeder, R.G. (1987). Binding of transcription factor TFIID to the major late promoter during in vitro nucleosome assembly potentiates subsequent initiation by RNA polymerase II. Cell *51*, 613–622.

Yu, T.Y., Kao, Y.W., and Lin, J.J. (2014). Telomeric transcripts stimulate telomere recombination to suppress senescence in cells lacking telomerase. Proc. Natl. Acad. Sci. U S A *111*, 3377–3382.

Zhang, Y., Liu, T., Meyer, C.A., Eeckhoute, J., Johnson, D.S., Bernstein, B.E., Nusbaum, C., Myers, R.M., Brown, M., Li, W., and Liu, X.S. (2008). Model-based analysis of ChIP-Seq (MACS). Genome Biol. *9*, R137.

Zhang, Z.Z., Pannunzio, N.R., Hsieh, C.L., Yu, K., and Lieber, M.R. (2015). Complexities due to single-stranded RNA during antibody detection of genomic rna:dna hybrids. BMC Res. Notes *8*, 127.

Zheng, S., Vuong, B.Q., Vaidyanathan, B., Lin, J.Y., Huang, F.T., and Chaudhuri, J. (2015). Non-coding RNA generated following lariat debranching mediates targeting of AID to DNA. Cell *161*, 762–773.

STAR★METHODS

KEY RESOURCES TABLE

REAGENT or RESOURCE	SOURCE	IDENTIFIER
Antibodies		
Mouse monoclonal anti-TFIIA-γ	Santa Cruz Biotechnology	Cat# sc-374483; RRID: AB_10988064
Mouse monoclonal anti-TFIIB	Santa Cruz Biotechnology	Cat# sc-271736; RRID: AB_10709889
Mouse monoclonal anti-TAF II p250 (TFIID)	Santa Cruz Biotechnology	Cat# sc-735; RRID: AB_671202
Mouse monoclonal anti-TFIIE-β	Santa Cruz Biotechnology	Cat# sc-137000; RRID: AB_2114531
Mouse monoclonal anti-TFIIF RAP30	Santa Cruz Biotechnology	Cat# sc-136408; RRID: AB_10647770
Mouse monoclonal anti-TFIIH p89	Santa Cruz Biotechnology	Cat# sc-271500; RRID: AB_10649033
Mouse monoclonal anti-TBP	Diagenode	Cat# C15200002
Mouse monoclonal anti-RNase H1	Abcam Ltd	Cat# ab56560; RRID: AB_945244
Rabbit polyclonal anti-RNase H2A	Abcam Ltd	Cat# ab83943; RRID: AB_1861175
Mouse monoclonal anti-BrdU	Sigma Aldrich	Cat# B2531; RRID: AB_476793
Rabbit polyclonal anti-TOP1	Bethyl	Cat# A302-589A; RRID: AB_2034865
Rabbit polyclonal anti- IBP160 (or AQR)	Bethyl	Cat# A302-547A; RRID: AB_1998964
Rabbit polyclonal anti-SETX	Bethyl	Cat# A301-105A; RRID: AB_2186221
Mouse monoclonal anti-RNA:DNA hybrids, (S9.6)	Proudfoot Lab	N/A
Rabbit polyclonal anti-GFP	Abcam	Cat# ab290; RRID: AB_303395
Bacterial and Virus Strains		
E. coli XL1Blue	Proudfoot Lab	N/A
Chemicals, Peptides, and Recombinant Proteins		
HeLa (human) cell extract	Ipracell	Cat# CC-01-20-50
Protein G Dynabeads	ThermoFisher Scientific	Cat# 10003D
Protein A Dynabeads	ThermoFisher Scientific	Cat# 10001D
Dynabeads sheep anti mouse IgG	ThermoFisher Scientific	Cat# 11031
Critical Commercial Assays		
Ribo-Zero Gold rRNA removal kit (H/M/R)	Illumina	Cat# MRZG12324
EpiMark bisulfite conversion	NEB	Cat# E3318S
QuikChange II XL Site directed mutagenesis	Agilent	Cat# 200521
Turbo DNase with inactivation buffer	Life Technologies	Cat# AM1907
NEBNext Ultra II Directional RNA library prep kit for illumina	NEB	Cat# E7760S
MaxiScript T7/T3 transcription kit	ThermoFisher Scientific	Cat# AM1326
Deposited Data		
Raw sequencing data	This paper	GEO: GSE87607
Re-analyzedR-ChIP data	Chen et al., 2017	GEO: GSE97072
Re-analyzed DRIPc-seq data	Sanz et al., 2016	GEO: GSE70189
Re-analyzed TFIIB data	Liang et al., 2015	GEO: GSE71848
Re-analyzed DNase-seq data		The ENCODE Project Consortium
Re-analyzed TBP ChIP-seq data	The ENCODE Project Consortium	GEO: GSM935606
Re-analyzed Pol II ChIP-seq data	The ENCODE Project Consortium	GEO: GSM935395
Re-analyzed MNuc-seq data for H3k4me3	Nojima et al., 2018b	GEO: GSM2976692
Experimental Models: Cell Lines		
Human HeLa	Proudfoot lab	N/A

(*Continued on next page*)

Continued		
REAGENT or RESOURCE	SOURCE	IDENTIFIER
Oligonucleotides		
Primers for plasmid pCIUBU construction and strand specific RNA transcript, see Table S1	This paper	N/A
Primers for RT-qPCR for *in vitro* transcription, see Table S2	This paper	N/A
Sequences of DNA or RNA oligonucleotides used for S9.6 validation on dot blot validation, see Table S3	This paper	N/A
Primers for RT-qPCR of antisense transcript, Br-U NRO and DIP validations, see Table S4	This paper	N/A
Primers for PIC validation, see Table S5	This paper	N/A
siRNAs used in this study, see Table S6	This paper	N/A
Recombinant DNA		
pCI-UraACTBUra	This paper	pCIUBU
Software and Algorithms		
Cutadapt(v1.9.1)	Martin, 2011	http://cutadapt.readthedocs.io/en/stable/installation.html
Tophat(v2.1.0)	Kim et al., 2013; Trapnell et al., 2010	http://ccb.jhu.edu/software/tophat/index.shtml
Cufflinks(v2.2.0)	Trapnell et al., 2010	http://cole-trapnell-lab.github.io/cufflinks/getting_started/
bedtools (v2.25.0)	Quinlan and Hall, 2010	http://bedtools.readthedocs.io/en/latest/content/installation.html
Bowtie2 (v2.2.5)	Langmead and Salzberg, 2012	http://bowtie-bio.sourceforge.net/bowtie2/index.shtml
SAMtools (v1.6)	Li et al., 2009	http://www.htslib.org/
Picard (v1.131)		http://broadinstitute.github.io/picard/
Deeptools (v2.5.3)	Ramírez et al., 2016	http://deeptools.readthedocs.io/en/latest/index.html

LEAD CONTACT AND MATERIALS AVAILABILITY

Further information and requests for resources and reagents should be directed to the lead contact, Nicholas Proudfoot (nicholas.proudfoot@path.ox.ac.uk).

EXPERIMENTAL MODEL AND SUBJECT DETAILS

HeLa cells were maintained in high glucose Dulbecco's Modified Eagle's Medium (DMEM) with 10% fetal bovine serum (FBS).

METHOD DETAILS

In vitro R-loop formation and transcription assay

Genomic region of β-actin terminator (Skourti-Stathaki et al., 2014) was PCR amplified and inserted into the *URA3* gene from *S. cerevisiae*. This was cloned into pCI vector at the BamHI/BglII site (so lacking CMV and T7 promoters), to generate pCIUBU. T7 promoted S and T3 AS transcripts of the β-actin terminator region were synthesized with α-^{32}PUTP using MAXIscript (Thermo-Fisher Scientific). PCR amplicons were used as templates by simple amplification of T7 or T3 promoter primers onto 5′ or 3′ ends of the β-actin terminator region respectively. Primers employed to generate pCIUBU and the ssRNAs are listed in Table S1. Synthetic R-loops were generated by modifying a previous protocol (Lee and Clayton, 1996). Briefly, 10 pmol of pCIUBU was mixed with 10 pmol of radio-labeled strand specific transcript in a 100 ul reaction mixture in R-loop buffer (62% formamide, 400 mM NaCl, 25 mM HEPES [pH7.5], 1.25 mM EDTA). Following incubation at 92°C for 2 min then at 62°C for 4 hr, the temperature was slowly reduced (1°C every 8.5 min) to 42°C, held at 42°C for 6 hr and then slow cooled to 37°C, 1°C per hr. The mixture was finally incubated at room temperature for 1 hr. Where indicated, the mixture was further digested with RNase H (Promega), RNase A (Sigma) or RNase T1 (Invitrogen). R-loops were detected by 0.8% agarose gel electrophoresis followed by autoradiography. R-loops were purified by first removal of formamide by Sephadex G-50 spin column (GE Healthcare) followed by selection of R-loop containing plasmid by

immobilised S9.6 antibody Dynabeads. *In vitro* transcription was performed on beads with selected R-loop plasmid in transcription buffer (40 mM HEPES-KOH pH7.9), 0.5 mM DTT, 3 mM $MgCl_2$, 60 mM KCl, 5 mM phosphocreatine, 0.5% polyvinyl alcohol, 12% glycerol, 500 uM NTP, 90 ug HeLa nuclear extract (Ipracell) for 1 hr. HeLa nuclear extract was RNase H1 and H2A immunodepleted using antibodies (Abcam) at 1 ug antibody per 10 ug nuclear extract. These were incubated for 2 hr at room temperature followed by removal of the precipitates with Dynabeads (ThermoFisher Scientific). RNA was extracted with TRIzol reagent (Ambion) and precipitated with isopropanol. Quantitation of the *in vitro* transcribed RNA was analyzed using RT-qPCR with primers as listed (Table S2).

Bisulfite conversions on *in vitro* plasmid R-loop

Exposed ssDNA on R-loop plasmid was detected by subjecting 1 μg of R-loop plasmid to bisulfite conversion (EpiMark Bisulfite conversion kit, NEB), essentially following manufacturer's instructions with the exception that the native bisulfite treatment was performed overnight at 37°C. PCR was subsequently carried out with forward and reverse primers corresponding to the 5′ and 3′ ends of *S. cerevisiae URA3* (listed in Table S2) before cloning using Agilent PCR Strataclone.

Cell transfection of siRNA and plasmids

RNAi was performed with Lipofectamine RNAiMax (Life technologies), delivered at 30 nM final concentration. siRNAs used are listed (Table S3). Lipofectamine 2000 (Life technologies) was employed to deliver RNase H1 overexpression plasmid (pEGFP-M27-H1) (Cerritelli et al., 2003) or as we have renamed it pRNH1-GFP, pGFP (pMAXGFP, Lonza), $pRNH1^{D210N}$-GFP and $pRNH1^{WKKD}$-GFP plasmids. All transfections employed 6-7 × 10^6 HeLa cells. Site directed mutagenesis was used to generate both $pRNH1^{D210N}$-GFP and $pRNH1^{WKKD}$-GFP (W43A, K59A, K60A and D210N) mutants using QuikChange II Site-Directed Mutagenesis Kit according to manufacturer specifications (Agilent).

Total RNA and chromatin-associated RNA (ChrRNA) library preparation

Total RNA was isolated with TRIzol (Ambion) according to manufacturer instructions. The procedure used for isolating chromatin-associated RNA is as described (West et al., 2008). HeLa cells were transfected with pRNH1-GFP (Cerritelli et al., 2003) and pGFP (Lonza) plasmids for 36 hr followed by FACS sorting to select transfected cells. Library preparations of chromatin associated RNA fraction begins by rRNA depletion with Ribo-Zero gold rRNA-removal kit Human/Mouse/Rat (Illumina MRZG12324) from 5 ug of ChrRNA. The resulting 100 ng of ribosomal depleted ChrRNA was used to make the libraries according to the manual of NEBNext Ultra II directional RNA library prep kit for Illumina (New England Biolabs). Libraries were sequenced on Illumina NEXTseq 550 with 42bp paired end reads.

RNA quantitation by RT-qPCR

HeLa cell derived chromatin-associated RNA or *in vitro* transcribed RNA was extracted with TRIzol and analyzed using gene specific primers by RT-qPCR. Primers used for RT-qPCR are presented in Tables S2 and S4.

Br-UTP nuclear run-on analysis

The Br-UTP NRO was carried out as previously described (Gromak et al., 2013), followed by RT-qPCR analysis as described above. The primers used are listed in Table S4.

Western blot

Cell extracts were prepared in 15 mM HEPES, pH 7.5, 0.25 M NaCl, 0.5% NP-40, 10% glycerol, 1 × protease inhibitor (Roche) and 1 mM PMSF. Proteins were separated by 4%–12% Tris-glycine SDS-PAGE and transferred to nitrocellulose (0.45 μM, Amersham Biosciences), and protein detection was carried out by standard western blot techniques. Anti-TOP1, AQR, SETX (Bethyl Laboratories), and Tubulin (Sigma) were used as primary antibodies in this study. Secondary antibodies were anti-mouse (A9044; Sigma) and anti-rabbit (A0545; Sigma). Signals were detected using ECL kit (GE Healthcare).

S9.6 validation by dot blot western

The specificity of S9.6 antibody binding was validated by dot blot analysis. Mixtures of RNA:DNA, DNA:DNA, RNA:RNA, ssRNA, ssDNA and RNA G-quadruplexes in KCl or in LiCl oligonucleotides were blotted on a Hybond-N+ nylon transfer membrane. The membrane was then blocked overnight in 5% milk, followed by a two hr incubation with the primary S9.6 antibody at 4°C before being washed with 1% milk. Secondary anti-mouse antibody (A9044; Sigma) was used before washing with 1% milk. Signals were detected using ECL kit (GE Healthcare). The oligonucleotide sequences used for this are provided (Table S3).

5′ RACE

5′ RACE was carried out using the 5′/3′ RLM-RACE kit (Ambion) and 5′/3′ RACE kit (TAKARA) according to manufacture instructions.

RNA specific RR-ChIP-seq

RR-ChIP-seq procedure was modified from the previously described R-ChIP method (Chen et al., 2017). Briefly, chromatin immunoprecipitation (ChIP) was performed on HeLa cells expressing $pRNH1^{D210N}$-GFP, $pRNH1^{WKKD}$-GFP or pRNH1-GFP. Approximately

1 × 10^7 HeLa cells were crosslinked with 1% formaldehyde for 15 min at 37°C before 0.125M glycine was added to quench the residual formaldehyde for a further 5 min. Petri dishes were washed twice with cold PBS before cells were scraped off and lysed in 400 μL of cell lysis buffer (10 mM Tris-HCl pH8.0, 85 mM KCl, 0.5% NP-40 and 1xComplete) for 10 min on ice. Isolated nuclear pellets were resuspended in 400 μL of nuclear lysis buffer (25 mM Tris-HCl pH8.0, 0.5% SDS, 5 mM EDTA and 1xComplete) and incubated on ice for 10 min before sonication (Bioruptor) to shear chromatin to 200-500 bp. 5% chromatin fragments were saved for total input and the remaining immunoprecipitated with magnetic beads conjugated with anti-GFP antibody (Abcam) overnight in IP buffer (10 mM Tris-HCl pH8.0, 5 mM EDTA, 0.5% Triton X-100 and 0.15 M NaCl) at 4°C. The beads (with the IPed DNA) were washed with 1 mL of buffer A (20 mM Tris-HCl pH8.0, 2 mM EDTA, 0.05% SDS, 1% Triton X-100 and 0.165 M NaCl) once, 1 mL of buffer B (20 mM Tris-HCl pH8.0, 2 mM EDTA, 0.05% SDS, 1% Triton X-100 and 0.5 M NaCl) once, 1 mL of buffer C (10 mM Tris-HCl pH8.0, 1 mM EDTA, 1% NP-40, 1% Sodium Deoxycholate and 0.25 M LiCl) once and then 1 mL of buffer D (10 mM Tris-HCl pH8.0 and 1 mM EDTA) twice. The chromatin complex was eluted in 300 μL of buffer E (1% SDS, 0.1 M $NaHCO_3$ and 0.5 M NaCl) at 65°C for 20 min before further incubation at 65°C overnight to reverse crosslink. 0.3mg/mL Proteinase K was added and incubated 45°C for 2 hr. The nucleic acids were extracted by phenol/chloroform (Sigma) and precipitated with glycogen before resuspension in nuclease free water. The hybrid fragments were then incubated at 90°C for 3 min (to separate R-loop strands), and quickly cooled to 4°C before subjecting to DNase I treatment. RNA moiety of the R-loop was extracted with TRIzol and precipitated with isopropanol and glycogen. These were used to make the libraries according to the manual of NEBNext Ultra II Directional RNA Library Prep kit for Illumina (New England Biolabs). Libraries were sequenced on Illumina NEXTseq 550 with 42bp paired end reads.

DIP and RNA specific RNA-DNA hybrid immunoprecipitation analysis (RDIP-seq)

DIP and RDIP-seq experiments were modified from previous method (Skourti-Stathaki et al., 2011). Briefly, nuclei were isolated from transfected HeLa cells from an 80% confluent 10 cm^2 plate. Following nuclear lysis, nuclear extracts were incubated with 30 μg of proteinase K (Roche) at 37°C overnight, and genomic DNA was isolated and quantitated. Genomic nucleic acids were pre-treated with RNase I (Promega) at 2 U per 100 ug nucleic acids for 15 min at 37°C) to reduce noise as S9.6 antibody can weakly detect nonspecific RNA conformations (Zhang et al., 2015). These were then sonicated (Bioruptor) to 200-300bp. Half of fragmented nucleic acids were treated with RNase H (Roche) at 10U per 100 g nucleic acids at 37°C overnight. Both RNase H treated and untreated samples were subjected to S9.6 antibody immunoprecipitation overnight. Hybrids were enriched by immuno-magnetic precipitation with M-280 sheep anti-mouse IgG Dynabeads (ThermoFisher Scientific). They were then extracted by phenol/chloroform (Sigma) and precipitated in the presence of glycogen before resuspension in nuclease free water. Here, samples can be removed for DIP-qPCR analysis where specific primers were tested (Table S4). For RDIP-seq, samples were then incubated at 90°C for 3 min and quick cooled to 4°C before subjecting to DNase I treatment. RNA moiety of the R-loop was then extracted with TRIreagent (Sigma) and precipitated with isopropanol and glycogen. Libraries were prepared with the NEBNext Ultra II Directional RNA Library Prep Kit for Illumina (NEB) according to the manufacturer's guidelines. Libraries were sequenced on an Illumina NEXTseq 550 with 75 bp single end reads.

Chromatin CAP-seq (ChrCAP-seq)

ChrCAP-seq experiments were modified from a previous method (Pelechano et al., 2015). Briefly, 50ug of FACs sorted chromatin associated RNA were treated with Turbo DNase I (Life technologies) according to manufacturer's instructions. RNA was recovered after acid phenol treatment. Sequential order of enriching for capped RNA started by first, the removal of 5′P RNA. RNA samples were treated with 1U Terminator 5′P dependent exonuclease (Epicenter, TER51020) before recovering the RNA through acid phenol treatment. The samples were then subjected to 30U Calf Intestine Phosphatase treatment (NEB, M0290S) to further remove 5′P and 5′PPP uncapped RNA before recovering the cap-enriched RNA by acid phenol treatment. The removal of 5′cap from RNA was performed using Cap-Clip Acid pyrophosphatase (CellScript, C-CC15011H). The resulting 5′P RNA enables single stranded RNA ligation of adaptor rp5: (5′CTTTCCCTACACGACGCTCTTCCGATrCrUrNrNrNrNrNrNrNrN-3′) with T4 RNA ligase (NEB M0204S). The resulting RNA was then recovered by acid phenol treatment and used for library preparation. These RNA libraries were prepared with the NEBNext Ultra II Directional RNA Library Prep Kit for Illumina (NEB) according to the manufacturer's guidelines. Libraries were sequenced on an Illumina NEXTseq 550 with 150 bp single end reads.

Chromatin Immunoprecipitation (ChIP) of GTFs

Approximately 1x10^7 HeLa cells (in 10 mL DMEM) were crosslinked with 1% formaldehyde at 37°C for 15 min with gentle shaking before 0.125M glycine was added to quench the residual formaldehyde for a further 5 min. Cells were washed twice with cold PBS before centrifugation at 1,400 rpm for 5 min to collect cells into 10 mL tube (Nunc). The washed cells were lysed with 400 μL of cell lysis buffer (10 mM Tris-HCl pH8.0, 85 mM KCl, 0.5% NP-40 and 1xComplete) and incubated on ice for 10 min. They were then centrifuged at 2,400 rpm for 5 min to remove the supernatant (cytoplasm fraction) before resuspension of nuclear pellets in 400 μL of nuclear lysis buffer (25 mM Tris-HCl pH8.0, 0.5% SDS, 5 mM EDTA and 1xComplete) and incubation on ice for 10 min. Cell suspensions were sonicated for 15 min (medium power, 30 s on-off repeats). To collect 400 μL of the supernatant as a soluble chromatin fraction, sonicated nuclei were centrifuged at 13,000 rpm for 10 min before 8-fold dilution with IP dilution buffer (10 mM Tris-HCl pH8.0, 5 mM EDTA, 0.5% Triton X-100 and 0.15 M NaCl). 5% Input was collected before the sonicated chromatin suspensions were immuno-precipitated with magnetic beads conjugated with anti-TFIIA, TFIIB, TFIID, TFIIE, TFIIF, TFIIH, TBP and IgG (as a

negative control) overnight at 4°C. IPed DNA was washed with 1 mL of buffer A (20 mM Tris-HCl pH8.0, 2 mM EDTA, 0.05% SDS, 1% Triton X-100 and 0.165 M NaCl) once, 1 mL of buffer B (20 mM Tris-HCl pH8.0, 2 mM EDTA, 0.05% SDS, 1% Triton X-100 and 0.5 M NaCl) once, 1 mL of buffer C (10 mM Tris-HCl pH8.0, 1 mM EDTA, 1% NP-40, 1% Sodium Deoxycholate and 0.25 M LiCl) once and then 1 mL of buffer D (10 mM Tris-HCl pH8.0 and 1 mM EDTA) twice. IPed beads were incubated with 0.01 mg/mL RNase A (Ambion) in 300 μL of buffer E (1% SDS, 0.1 M $NaHCO_3$ and 0.5 M NaCl) at 65°C for at least 4 hr. After RNase treatment, 30 μL of 10x Proteinase K mixture (200 mM Tris-HCl pH 6.5, 150 mM EDTA and Proteinase K 0.3 mg/mL) were added and then incubated 45°C for 2 hr. DNA fragments were purified using phenol/chloroform (pH 7.0) and ethanol precipitation. qPCR was carried out to determine the occupancy of the general transcription factors. The primers are listed in Table S5.

QUANTIFICATION AND STATISTICAL ANALYSIS

The number of n biological replicates is provided within each figure legend. Statistical p values were calculated using two-tailed Student's t tests with two sample assuming unequal variances. The error bars denote SEM, the center values denote mean.

Bioinformatic analysis

hg19/GRCh37 was used as a reference genome. Gene boundaries were obtained from ENSEMBL (GRCh37.75; (Flicek et al., 2014). All genes were taken from the most 5′ TSS to the most 3′ TES. A set of non-overlapping protein-coding genes was achieved by retaining genes showing no transcript overlap (within 2 kb) with other (protein coding or non-coding) genes in the genome according to the ENSEMBL annotation. Only genes longer that 2 kb were considered for this study. PROMPTs, eRNAs, and lincRNA annotation was employed as previously described (Nojima et al., 2018b).

Data processing and visualization

ChrRNA-seq

Adaptors were trimmed using Cutadapt (Martin, 2011) in paired-end mode discarding reads with less than 20 bases. Resulting paired-end reads for each sample were then mapped to human genome reference assembly GRh37/hg19 (build 37.2, February 2009) with Tophat v. 2.0.13 (Kim et al., 2013), https://ccb.jhu.edu/software/tophat/) and the parameters -g 1 -r 3000–no-coverage-search. Properly paired and mapped reads were obtained with SAMtools v. 1.2 ((Li et al., 2009), http://www.htslib.org/) using samflags 0x63, 0x93, 0x53, 0xA3. Number of reads mapped to each gene was normalized for length and total number of genome-aligned reads (RPKM) with Bedtools (genomecov –bg –scale). For genes with multiple isoforms, the expression levels were calculated using the length of the longest isoform. For data visualization, UCSC genome browser trackhubs were created by employing the UCSC bedGraphToBigWig tool (Kent et al., 2002).

RR-ChIP-seq

Raw reads from RR-ChIP-seq were demultiplexed using in-house Perl script and aligned to reference genome hg19/GRCh37 using bowtie2 (Langmead and Salzberg, 2012). Uniquely mapped reads with no mismatches were retained for further analysis. Plus and minus strand were assigned to mapped reads using SAMtools. RR-ChIP-seq peaks were called using MACS2 (Zhang et al., 2008) algorithm with default options. R-loop peaks with > 5 folds enrichment and with q-value < 0.05 were retained for subsequent analyses. This resulted in 27868 peaks. Strands were assigned to peaks by intersecting the called peaks to strand specific reads using bedtools (Quinlan and Hall, 2010). The RR-ChIP peak summit files generated by MACS2 were used for further downstream analysis.

RR-ChIP peaks were assigned to one of the following categories; TSS peaks spanned from 2kb upstream to 1kb downstream of TSS; TES peaks overlapped a window of 1kb upstream to 2kb downstream of annotated poly(A) site; Genebody peaks were the remaining genic region; AS peaks overlapped 2 kb upstream to 2 kb downstream of a gene but in reverse direction and Intergenic peaks show no overlap with any above-mentioned categories.

ChrCAP-seq

ChrCAP-seq reads (strand-specific, single-end, 160 bp) were mapped to the human genome (hg19) using Bowtie2 in via default local mode. SAMtools was used to filter non-redundant reads (-q 2). Bedtools was used to generate library-size normalized bedgraph files and trackhubs in the UCSC browser were generated with the UCSC bedGraphToBigWig tool. ChrCAP-seq clusters were identified using *findpeaks* function with '-style tss' from the HOMER software r (http://homer.salk.edu/homer/). This resulted in 20,417 ChrCAP-seq clusters.

Metagene profiles

To plot the distribution of RR-ChIP peaks relative to TSS and TES, bedtools closest function was used to obtain distance of peak summits from TSS and TES for D210 and WKKD samples. Peak density was subsequently plotted for both S and AS plotted using Matplotlib (Hunter, 2007) in Python.

TSS and TES profiles were obtained by plotting normalized read counts around annotated 3′end (TES plots) and 5′end (TSS plots) for sense strand relative to the direction of gene transcription using in-house Perl scripts. Graphs were plotted using Matplotlib (Hunter, 2007).

For data visualization in boxplot format, gene expression in Reads per Million mapped reads (RPM), was calculated for all non-overlapping genes. Sense gene expression are reads mapped to S direction of the gene and AS gene expression was calculated for reads mapped to reverse direction of a S gene.

For eRNA analysis, bedtools *closest* function was used to overlap RR-ChIP peaks to annotated eRNA regions. Peaks within a window of ± 500bp of annotated eRNA regions were considered as overlapping. Regions where the RPKM fold change upon RNase H1 overexpression was at least 1.5 times compared to the wild-type were considered as sensitive to RNase H1 overexpression.

RNase H1 sensitive capped RNA analysis, antisense expression was calculated for all ChrCAP-seq clusters to find regions that show RNase H1 overexpression sensitivity. Out of the in 20,417 ChrCAP-seq clusters scanned, 2160 were sensitive to RNase H1 overexpression. Effect of RNase H1 overexpression on the expression of ChrRNA-seq sense and antisense transcripts for these regions were then visualized using boxplot.

PICs correlation heatmap

Heatmap showing the correlation of RR-ChIP peaks in a +-1kb window with DNASE-seq, Pol II, TBP, TFIIB ChIP-seq and H3K4me3 mNuc-seq datasets was computed using Deeptools2.1.0. (Ramírez et al., 2016).

DATA AND CODE AVAILABILITY

The accession number for the genome-wide datasets reported in this paper is GEO: GSE87607. Public data analyses for R-ChIP-seq and DRIPc-seq were downloaded from NCBI Gene Expression Omnibus with the accession number GEO: GSE97072 and GEO: GSE70189 respectively. For comparison of ChrCAP-seq and 5′-GRO-seq, 5′-GRO-seq data was downloaded from GEO: GSE63872.

BigWig files for DNase-seq, PolII and TBP ChIP-seq data for HeLa-S3 cells were downloaded from the UCSC ENCODE ftp server. TFIIB data was downloaded from the GEO repository GEO: GSE71848. H3K4Me3 mNuc-seq data was downloaded from GEO: GSM2976692.

Molecular Cell, Volume *76*

Supplemental Information

R-Loops Promote Antisense Transcription across the Mammalian Genome

Sue Mei Tan-Wong, Somdutta Dhir, and Nick J. Proudfoot

Figure S1 (related to Figure 1)

A Efficiency of R-loop plasmid immunoselection

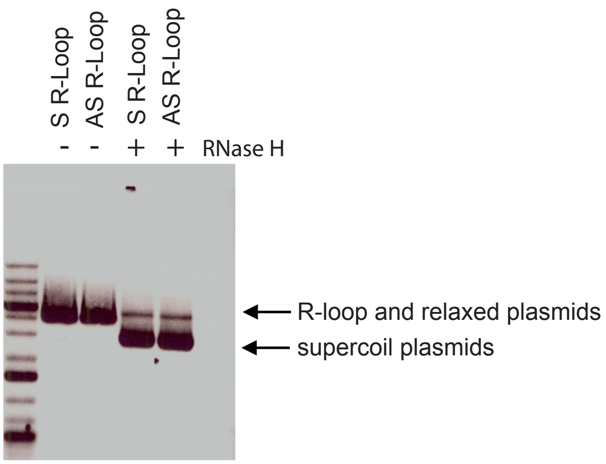

B Positions of capped AS transcripts

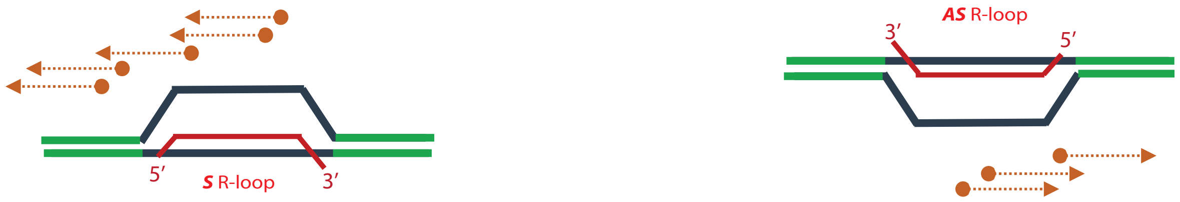

5′ end of AS transcript from S R-loop transcription

GTTGAGCATTAGGTCCCAAAATTTGTTTACTAAAAACACATGTGGATATCTTGACTGATTTTT
CCATGGAGGGCACAGTTAAGCCGCTAAAGGCATTATCCGCCAAGTACAATTTTTTACTCTTC
GAAGACAGAAAATTTGCTGACATTGGTAATACAGTCAAATTGCA**G**TACTCTGCGGGTGTATA
CAGAATAGCAGAATGGGCAGACATTACGAATGCACACGGTGTGGTGGGCCCAGGTATTGTT
AG**C**GGTTTGAAGCAGGCGGCAGAAGAAGTAACAAAGGAACCTAGAGGGGGACTATTTGGG
GGTGTCTGGCT**C**AGGTGCCATGCCTCACTGGGGCTGGTTGGCACCTGCATTTCCTGGAGTGG
GGCTGTCTCAGGGTAGCTGGG**C**ACGGTGTT**C**CCTTGAGTGGGGGTGTAGTGGGTGTTCCTA
GCTGCCACGCCTTTGCCTTCACCTATGGGACCTTTTGATGTTAGCAGAATTGTCATGCAAGGG
CTCCCTATCTACTGGAGAATATACTAAGGGTACTGTTGACATTGCGAAGAGCGACAAAGATT
TTGTTATCGGCTTTATTGCTCAAAGAGACATGGGTGGAAGAGATGAAGGTTACGATTGGTTG
ATTATGACACCCGGTGTGGGTTTAGATGACAAGGGAGACGCATTGGGTCAACAGTATAGAA
CCGTGGATGATGTGGTCTCTACAGGATCTGACATT

5′ end of S transcript from AS R-loop transcription

GTTGAGCATTAGGTCCCAAAATTTGTTTACTAAAAACACATGTGGATATCTTGACTGATTTTT
CCATGGAGGGCACAGTTAAGCCGCTAAAGGCATTATCCGCCAAGTACAATTTTTTACTCTTC
GAAGACAGAAAATTTGCTGACATTGGTAATACAGTCAAATTGCAGTACTCTGCGGGTGTATA
CAGAATAGCAGAATGGGCAGACATTACGAATGCACACGGTGTGGTGGGCCCAGGTATTGTT
AGCGGTTTGAAGCAGGCGGCAGAAGAAGTAACAAAGGAACCTAGAGGGGGACTATTTGGG
GGTGTCTGGCTCAGGTGCCATGCCTCACTGGGGCTGGTTGGCACCTG**C**ATTTC**C**TGGAGTGG
GGCTGTCTCAGGGTAGCTGGGCACGGTGTT**C**CCTTGAGTGGGGGTGTAGTGGGTGTTCCTA
GCTGCCACGCCTTTGCCTTCACCTATGGGACCTTTTGATGTTAGCAGAATTGTCATGCAAGGG
CTCCCTATCTACTGGAGAATATACTAAGGGTACTGTTGACATTGCGAAGAGCGACAAAGATT
TTGTTATCGGCTTTATTGCTCAAAGAGACATGGGTGGAAGAGATGAAGGTTACGATTGGTTG
ATTATGACACCCGGTGTGGGTTTAGATGACAAGGGAGACGCATTGGGTCAACAGTATAGAA
CCGTGGATGATGTGGTCTCTACAGGATCTGACATT

C Efficiency of R-loop promoter vs Adenovirus major late promoter

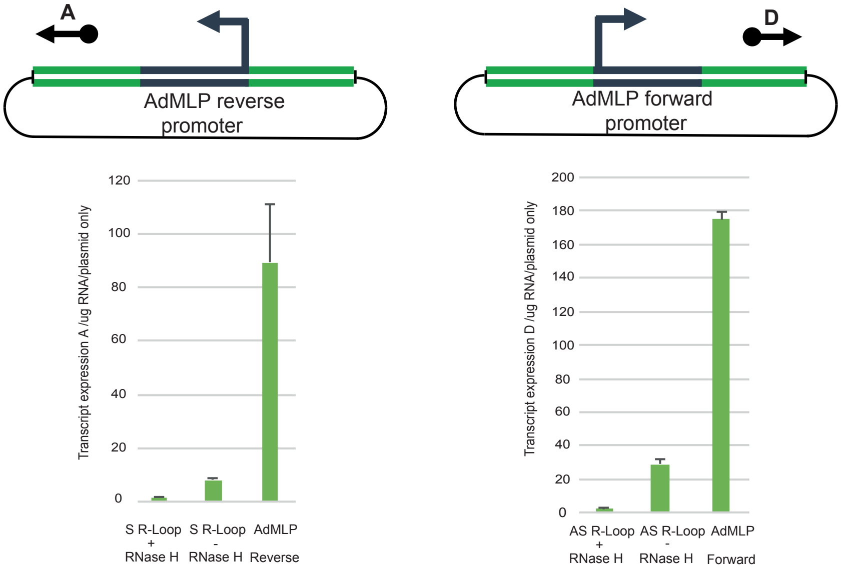

Figure S1 (related to Figure 1)
(A) Efficiency of R-loop plasmid immunoselection. Ethidium bromide stained agarose gel of S9.6 immunoselected R-loop plasmids, showing sensitivity to RNase H1 digestion.
(B) Sequence analysis of R-loop induced transcripts. 5'RACE shows different transcription start sites from R-loop promoter, depicted as bold orange nucleotides within the β-actin terminator region (blue nucleotides) or adjacent *URA3* regions (green nucleotides).
(C) Comparison of R-loop and adenovirus major late promoters *in vitro* transcription efficiencies. AdMLP promoter was inserted in place of the β-actin terminator R-loop region in an orientation specific manner within pCIUBU. *In vitro* transcription followed by RT-qPCR was carried out using the same *URA3* gene primer pairs as in Figure 1D. Data represented as mean ± SEM.

Figure S2 (related to Figure 2)

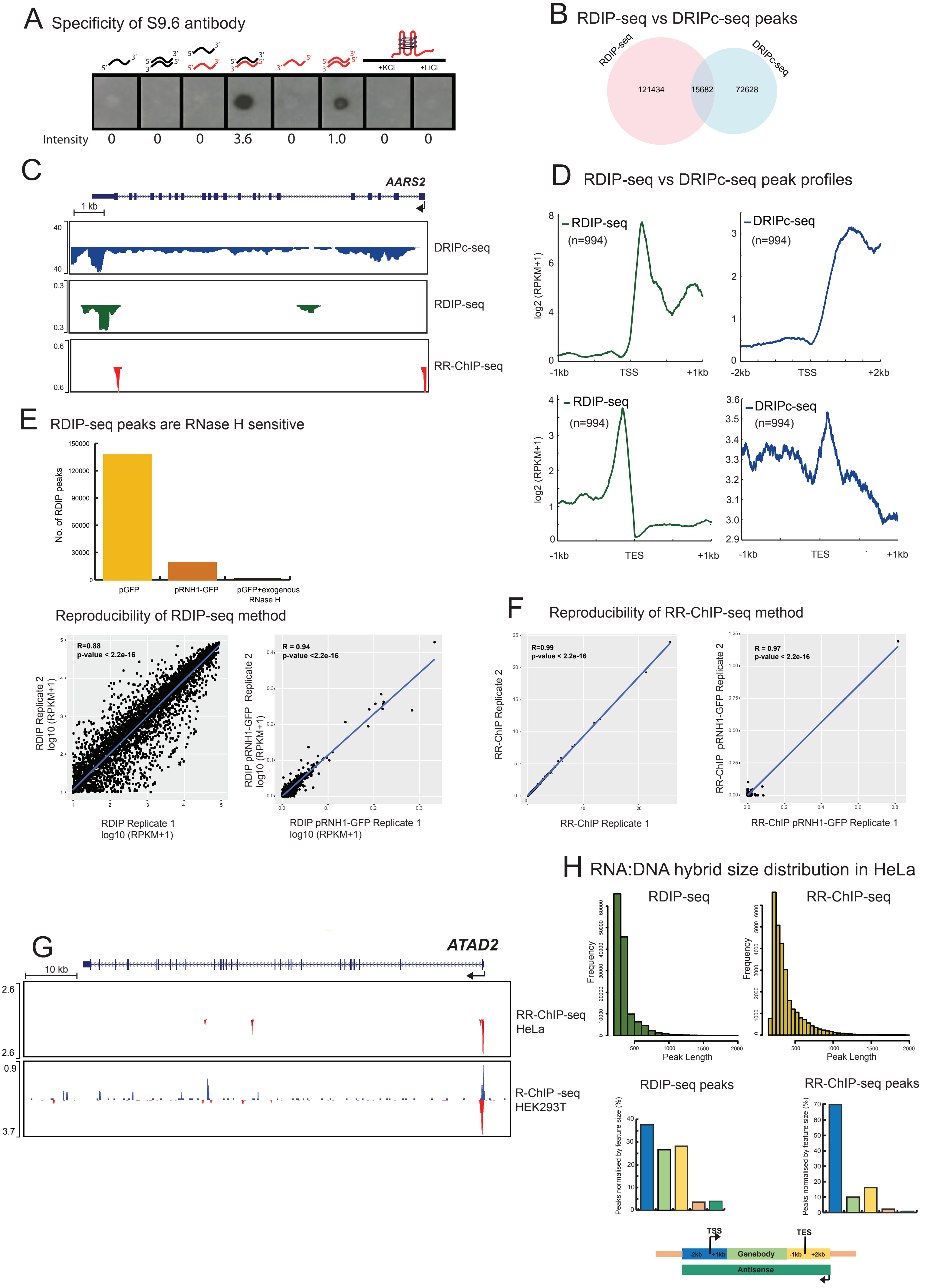

Figure S2 (related to Figure 2)

(A) Dot blot analysis of S9.6 antibody binding to different nucleic acid structures as indicated (Table S3). Intensities were measured with ImageStudio Lite program.

(B) Venn diagram showing the overlap of R-loop peaks identified by DRIPc-seq and RDIP-seq.

(C) *AARS2* R-loop profiles for DRIPc-seq, RDIP-seq and RR-ChIP-seq.

(D) Peak density plot showing distribution of RDIP-seq and DRIPc-seq relative to TSS and TES regions of protein coding genes.

(E) Graph showing the high proportion of RDIP-seq peaks that display RNase H sensitivity either by RNase H1 over-expression or by RNase H treatment following the immuno-precipitation step. Below shows a scatter plot of reproducibility between R-loop peaks detected by two RDIP-seq (Pearson correlation: R = 0.88). Transcripts of ≥1 RPKM were analysed. Similarly, for the two RDIP-seq with RNase H1 over-expression.

(F) Scatter plot showing high reproducibility between RR-ChIP-seq and RR-ChIP-seq with RNase H1 over-expression replicates (Pearson correlation: R = 0.99 and 0.97 respectively).

(G) *ATAD2* RR-ChIP-seq vs R-ChIP-seq peak profiles.

(H) Size distribution of RDIP-seq and RR-ChIP-seq peaks in HeLa cells. Level of RDIP-seq and RR-ChIP-seq peaks present at different gene regions, corrected for genomic feature size (lower diagram).

Figure S3 (related to Figure 3)

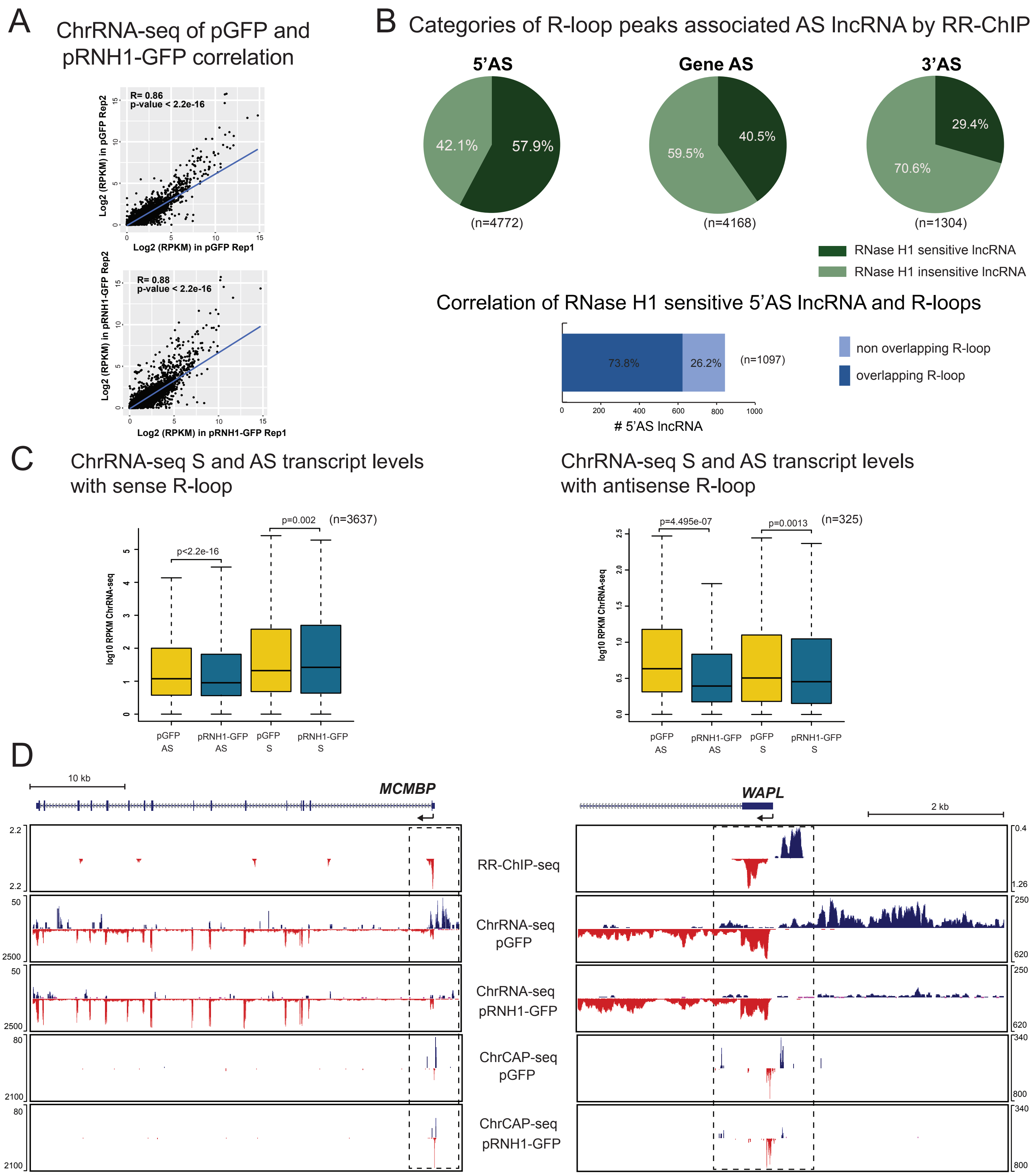

Figure S3 (related to Figure 3)

(A) Scatterplot of gene expression showing reproducibility (Pearson correlation: R = 0.86 and 0.88 respectively) between the two ChrRNA-seq datasets for pGFP and pRNH1-GFP over-expression. Transcripts of ≥1 RPKM were used.

(B) Pie diagrams showing total numbers of RR-ChIP peaks (+/-250 bp from lncRNA) for the three AS categories. % of these R-loop associated lncRNA that display RNase H sensitivity is indicated. Stacked bar chart showing correlation between RNase H1 sensitive 5'AS lncRNA and associated pRNH1-GFP sensitive R-loop peaks. 73.8% of RNase H1 sensitive 5'AS lncRNA has a corresponding RNase H1 sensitive R-loop overlap.

(C) Box plots showing range of S gene and AS lncRNA transcript levels associated with sense R-loops (left hand side) or antisense R-loops (right hand side). *P*-values were computed using Wilcoxon test.

(D) *MCMBP* and *WAPL* ChrRNA-seq versus ChrCAP-seq profiles +/- RNase H1 over-expression. RR-ChIP-seq profile also shown.

Figure S4 (related to Figure 4)

Further eRNAs

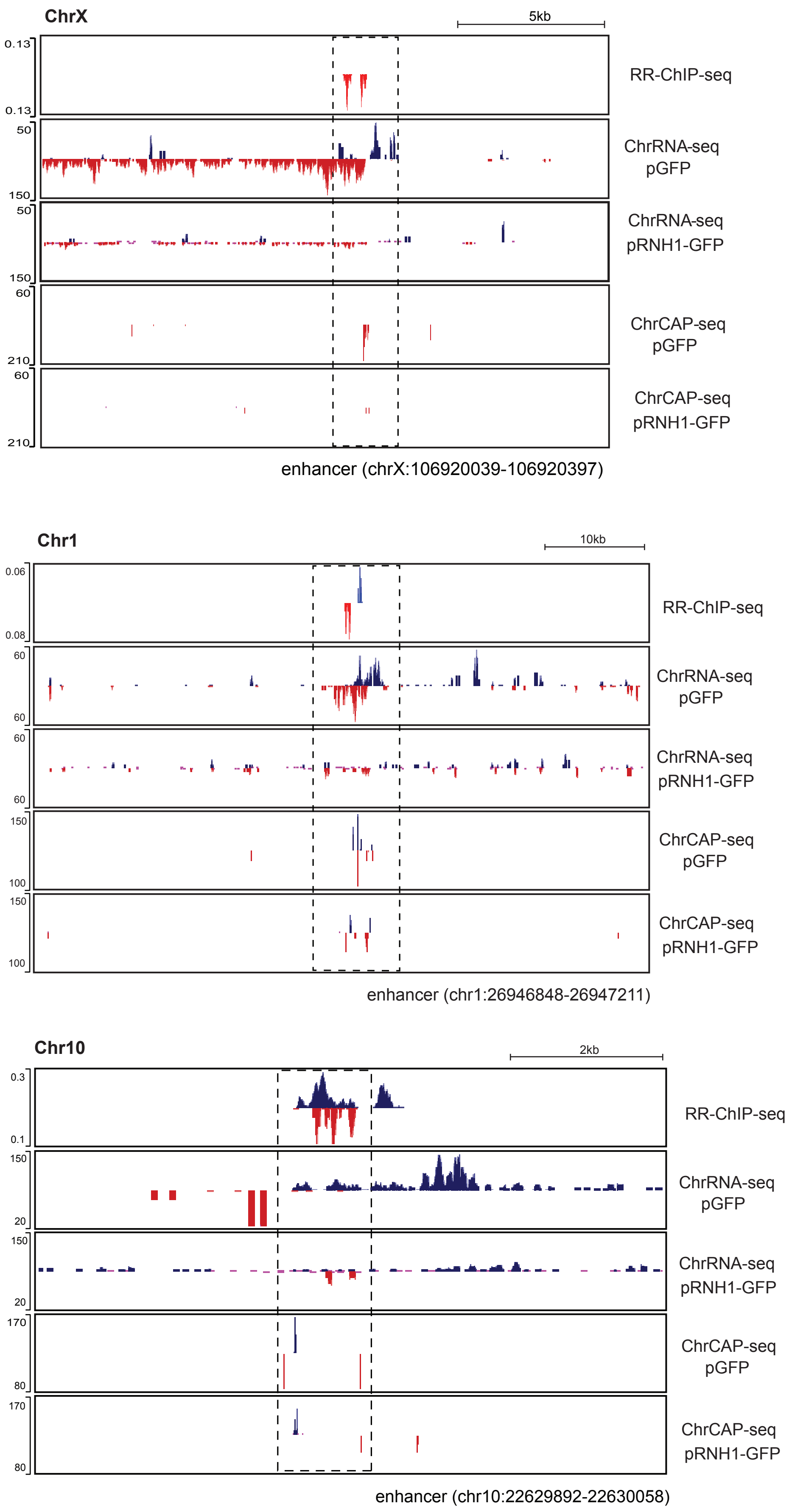

Figure S4 (related to Figure 4)
Three separate enhancer-derived eRNA profiles (from chromosome X, 1 and 10) based on ChrRNA-seq and ChrCAP-seq +/- RNase H1 over-expression versus RR-ChIP-seq profiles.

Figure S5 (related to Figure 5)

A ChrCAP-seq vs 5' GRO-seq comparison in HeLa

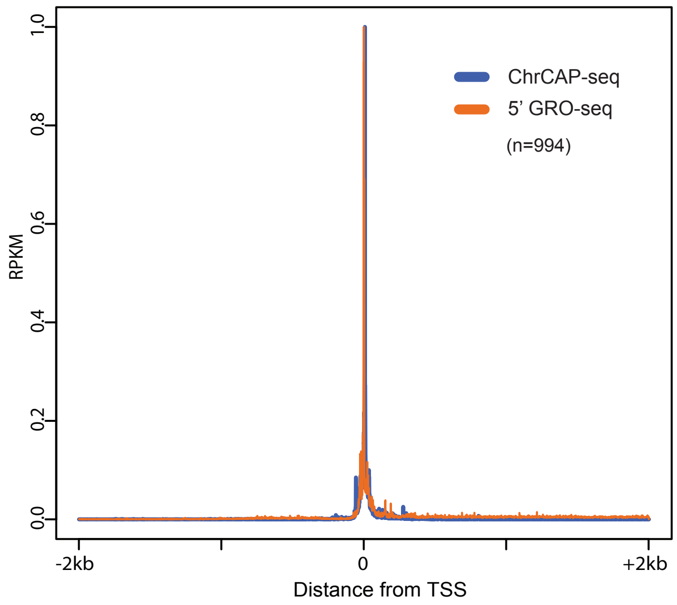

B Comparison example between ChrCAP-seq and 5' GRO-seq

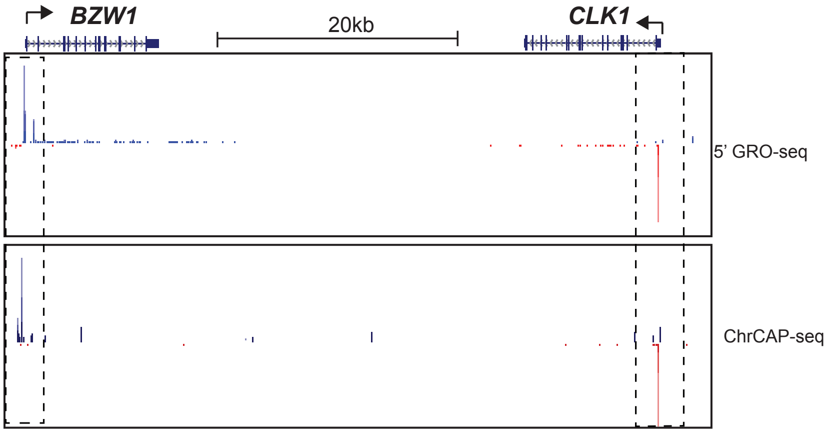

Figure S5 (related to Figure 5)
(A) Aggregation plots comparing signals for ChrCAP-seq and 5'GRO-seq from Hela cells across all transcription start sites (TSS) within a 2kb window on either side of TSS.
(B) ChrCAP-seq versus 5'GRO-seq profiles for *BZW1-CLK1* convergent gene locus.

Figure S6 (related to Figure 6)

A

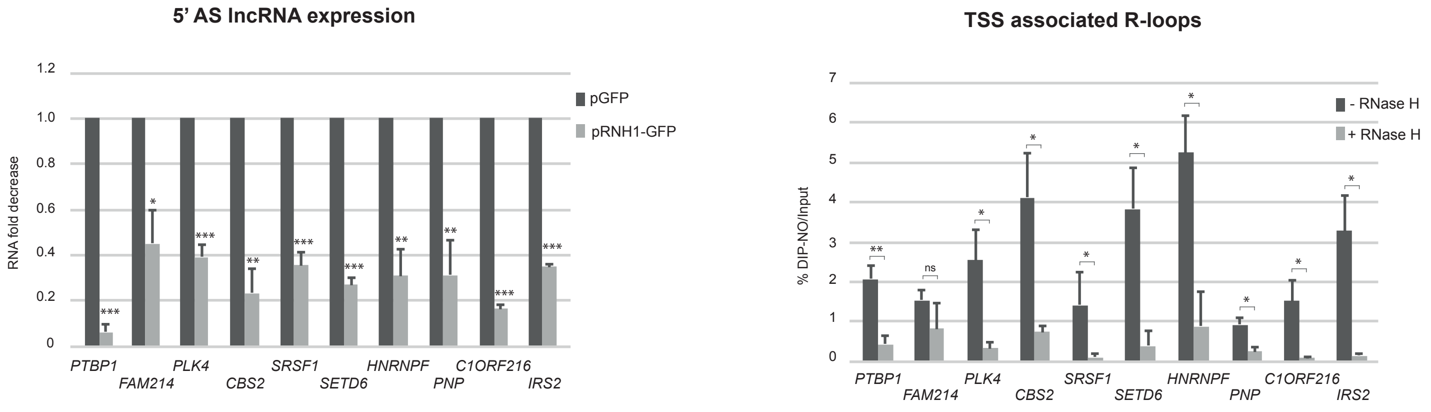

B

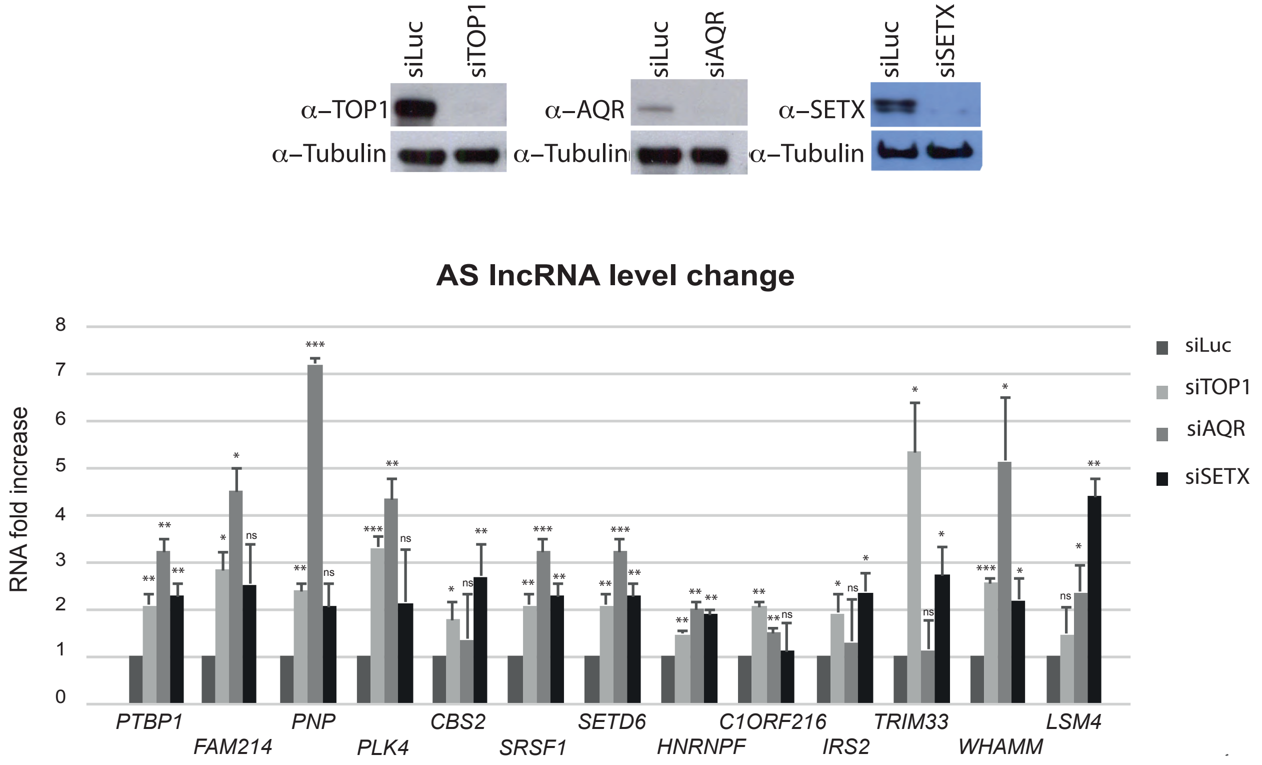

Figure S6 (related to Figure 6)

(A) RT-qPCR analysis of specific genes validating expression of RNase H1 sensitive antisense transcripts, identified by ChrRNA-seq. Chromatin RNA fraction from HeLa cells with or without RNase H1 over-expression analyzed using gene specific primers for RT reaction (Table S4). Relative expression values were normalized to 18S RNA. Data represented as mean ± SEM (left). DIP analyses (Skourti-Stathaki et al., 2011) of same regions as above, validating R-loop occupied regions. Data represented as mean ± SEM (right). (n=3, ***$p < 0.001$, **$p < 0.01$ and *$p < 0.05$, paired *t*-test).

(B) Enhanced R-loop formation increases antisense transcript levels. Top panels are western blots showing efficiency of TOP1, AQR and SETX knock downs following siRNA treatment. RT-qPCR results show that stabilizing R-loops by the various factor depletions increases antisense transcript levels for various genes. Data represented as mean ± SEM (n=3, ***$p < 0.001$, **$p < 0.01$ and *$p < 0.05$, paired *t*-test).

Figure S7 (related to Figure 7)

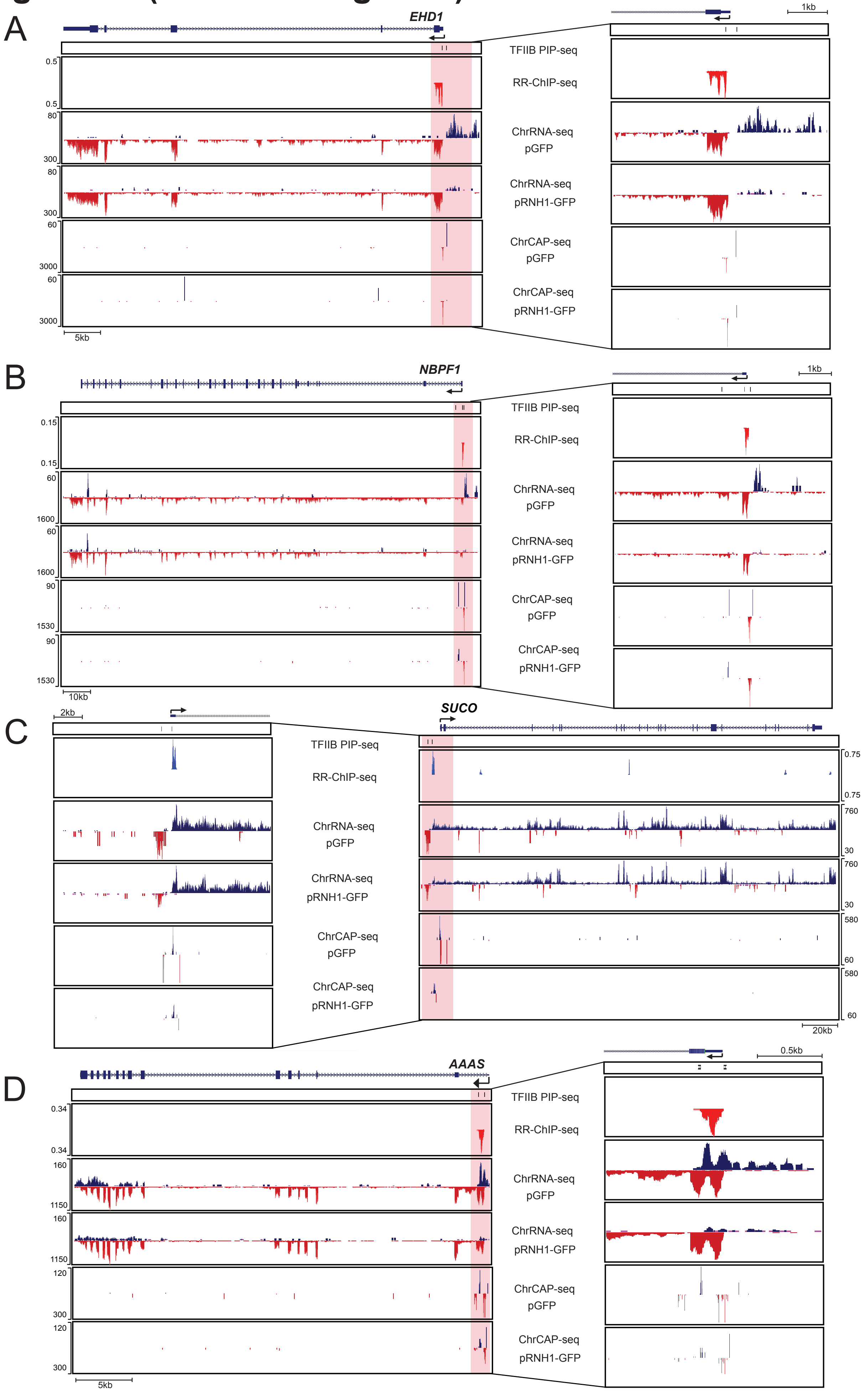

Figure S7 (related to Figure 7)
ChrRNA-seq and ChrCAP-seq profiles +/- RNase H1 over-expression compared to TFIIB-PIP-seq and RR-ChIP-seq for 4 selected genes as in Figure 7. Promoter regions are highlighted with pink shading and shown enlarged.

Table S1: Primers for plasmid pCIUBU construction and strand specific RNA transcript (related to STAR Methods)

PRIMER'S NAME	PRIMER'S SEQUENCES (5'-3')
H.s. ACTB terminator Forward	GGGACTATTTGGGGGTGTCTGGCT
H.s. ACTB terminator Reverse	CGATCCCATAGGTGAAGGCAAAGG
S. c. URA3 Forward	GATTCGGTAATCACCGAACAG
S. c. URA3 Reverse	GGAATCTCGGTCGTAATGATT
T7 *H.s.* ACTB terminator Forward	TTAATACGACTCACTATAGGGAGAGGACTATTTGGGGGTGTCTGGCT
T3 *H.s.* ACTB terminator Reverse	AATTAACCCTCACTAAAGGGAGACGATCCCATAGGTGAAGGCAAAGG

Table S2: Primers for RT-qPCR for *in vitro* transcription (related to STAR Methods)

PRIMER'S NAME	PRIMER'S SEQUENCES (5'-3')
Amplicon A Forward	ATAATGCCTTTAGCGGCTTA
Amplicon A Reverse	AACTTGTGTGCTTCATTGGA
Amplicon B Forward	AACTTGTGTGCTTCATTGGA
Amplicon B Reverse	ATAATGCCTTTAGCGGCTTA
Amplicon C Forward	GCTTCCCAGCCTGCTTTTCTG
Amplicon C Reverse	TAGATGACAAGGGAGACGCAT
Amplicon D Forward	TAGATGACAAGGGAGACGCAT
Amplicon D Reverse	GCTTCCCAGCCTGCTTTTCTG
Amplicon A RT primer	CATGCACGAAAAGCAAACAAAC
Amplicon B RT primer	TTGTACTTGGCGGATAATGCC
Amplicon C RT primer	ACACCCGGTGTGGGTTTAGAT
Amplicon D RT primer	CATCTTCTCAAATATGCTTCC

Table S3: Sequences of DNA or RNA oligonucleotides used for S9.6 validation on dot blot (related to STAR methods)

NUCLEOTIDE NAME	NUCLEOTIDE SEQUENCES
DNA 5' to 3'	GTTCAGAGTTCTACAGTCCGACGATC
DNA 3' to 5'	GATCGTCGGACTGTAGAACTCTGAAC
RNA 5'to 3'	rGUUrCrArGrArGUUrCUrArCrArGUrCrCrGrArCrGrAUrC
RNA 3'to 5'	rGrAUrCrGUrCrGrGrArCUrGUrArGrArArCUrCUrGrArArC
RNA G-quadruplex	$(GAGCUGGGGU)_4$

Table S4: Primers for RT-qPCR of antisense transcript, Br-U NRO and DIP validations (related to STAR methods)

PRIMER'S NAME	PRIMER'S SEQUENCES (5'-3')
TRIM33 for AS forward	CCCTCGAAGCCAGTTCCTTG
TRIM33 for AS reverse	GGCTACCACCCTTCCGTATG
TRIM33 for DIP forward	ATGCCCAGCTTTCCCTAACT
TRIM33 for DIP reverse	GGAAAGTGGACTGCATGGTT
WHAMM for AS forward	CAGACTCCTGAACAGGTTGC
WHAMM for AS reverse	GCTTACAAGAGGCATTCGG
WHAMM for DIP forward	GACGCGGTTTCGATTCTAGC
WHAMM for DIP reverse	AGGCTGTCAGGCTGCTCGTC
LSM4 for AS and DIP forward	TTGAGAGTCTCGATCTGTCG
LSM4 for AS and DIP reverse	TGATGGCACAAACCTGTAATC
MYOD1 forward	CGCTTTCCTTAACCACAAATC
MYOD1 reverse	AACACGGTCGTCATAGAAGT
SRSF1 for AS forward	CGAATAGACCGTGGCTGCTT
SRSF1 for AS reverse	GAGTGCTGACCCGAGCTATG
SRSF1 for DIP forward	GGTCCTCGAACTCAACGAAG
SRSF1 for DIP reverse	CGCATCTACGTGGGTAACTTA

PLK4 for AS forward	GAGGTCTCTTTTGATCCATACAA
PLK4 for AS reverse	AACACCGTTTACACCACTACC
PLK4 for DIP forward	GAAGGTGTCAGGGAGAACTTT
PLK4 for DIP reverse	CTGCCGAGTCTTTTCACCTC
SEDT6 for AS forward	CGGTCTGGGTGCAAGAACAA
SEDT6 for AS reverse	GCAACAAGGGGTCGAGATCA
SEDT6 for DIP forward	CTGCTTCCTGAGCTGGTGC
SEDT6 for DIP reverse	CACCACGAACAACAGCTCT
FAM214A for AS forward	CAGACTACAGGCATGCACAA
FAM214A for AS reverse	GCTCACTTTATGAATACAAGGCT
FAM214A for DIP forward	CAGGCTGTAAACACCTCCC
FAM214A for DIP reverse	GGCCAAGTTTACACAGAAACC
HNRNPF for AS forward	AAGTGGTGGGATTACAGGCG
HNRNPF for AS reverse	ATCCGTGACTGCTCCATTGC
HNRNPF for DIP forward	CTTTGCCGGAAGCCCTCCT
HNRNPF for DIP reverse	CTCCTTGGGCTTCTGTTTCCT
PTBP1 for AS forward	TCGAACTGTACGAGCTCCA
PTBP1 for AS reverse	GGCTCCATACAATCCAAGG
PTBP1 for DIP forward	GTTCCTGCTATTCCGGCGC
PTBP1for DIP reverse	CGACGTGCGACTCACCCGT
IRS2 AS and DIP forward	AAGCAGATCTTGCAGCCCA
IRS2 AS and DIP reverse	CGTTGAGGTAGTCCCCGTT
CBS AS and DIP forward	ATGTATCCGTCCAGGTGAGAT
CBS AS and DIP reverse	GGTCAAGACCGCAGGCGTG
CIORF216 AS forward	GTCCCCGTCCCTAGCTTGCT
CIORF216 AS reverse	CAGGTTATGGAGGAGAGAAGAG
CIORF216 DIP forward	CGACACGCCAGGACTAGGT
CIORF216 DIP reverse	CTACGCCGTGAGTCCGAGT
PNP AS forward	CTGGGAAGACCAAACGTTGC

PNP AS reverse	ATGTCTGGTTTCCCGGGC
PNP DIP forward	GCAAGGGATATAAGCCAGAGC
PNP DIP reverse	CTTCTCGATGAGCCTGCTCC

Table S5: Primers for PIC validation (related to STAR Methods)

PRIMER'S NAME	PRIMER'S SEQUENCES (5'-3')
TRIM33 sense forward	ATGCCCAGCTTTCCCTAACT
TRIM33 sense reverse	GGAAAGTGGACTGCATGGTT
TRIM33 antisense forward	CCCTCGAAGCCAGTTCCTTG
TRIM33 antisense reverse	GGCTACCACCCTTCCGTATG
EHD1 sense forward	CTGAACATACTGCCGGACAC
EHD1 sense reverse	GACCCTATAAAGGCCGCTCC
EHD1 antisense forward	CATTGGCTGATTCCAAATCTC
EHD1 antisense reverse	TGACTGGGTGTCTCGTGGAG
NBPF1 sense forward	CTTCGCGTAACTTCCCATTC
NBPF1 sense reverse	ATCCTGAGGAGCCAGTGG
NBPF1 antisense forward	CGTGTCAGGATAACCCAAGG
NBPF1 antisense reverse	AGCGATTTGGAGGGTCGAG
SUCO sense forward	GGCTGTTGAGAGGCGAGTA
SUCO sense reverse	GAGAATGTGACCGATAGTCCTAAG
SUCO antisense forward	CCTTCCCCACTTTAGGTGAAC
SUCO antisense reverse	CCAAGAATCCTCTCATCTGATT
AAAS sense forward	GGCTAGATTCGTATGCGGAC
AAAS sense reverse	CGCAGGAAGAAGCTTTGGA
AAAS antisense forward	GCGGGCTCTCATAGCTACTG
AAAS antisense reverse	GTTTGCCGACTGCAGACGT

Table S6: siRNA used in this study (related to STAR Methods)

siRNAs	Source	Identifier
siLuc (custom siRNA)	Sigma	Sequence (5'-3')<br>Sense: GAUUAUGUCCGGUUAUGUAUU<br>Antisense: [phos]UACAUAACCGGACAUAAUCUU
siTOP1 (human), ON-TARGETplus	Dharmacon (GE)	L-005278-00-0005
siAQR (human), ON-TARGETplus	Dharmacon (GE)	L-022214-01-0005
siSETX (human), ON-TARGETplus	Dharmacon (GE)	L021420-00-0005
